# Determining the best population-level alcohol consumption model and its impact on estimates of alcohol-attributable harms

**DOI:** 10.1186/1478-7954-10-6

**Published:** 2012-04-10

**Authors:** Tara Kehoe, Gerrit Gmel, Kevin D Shield, Gerhard Gmel, Jürgen Rehm

**Affiliations:** 1 Centre for Addiction and Mental Health (CAMH), Toronto, Canada; 2 Department of Statistics, University of Toronto, Toronto, Canada; 3 Dalla Lana School of Public Health (DLSPH), University of Toronto, Toronto, Canada; 4 Addiction Info Suisse, Lausanne, Switzerland; 5 Alcohol Treatment Centre, Lausanne University Hospital CHUV, Lausanne, Switzerland; 6 University of the West of England, Bristol, UK; 7 Institute for Clinical Psychology and Psychotherapy, Dresden University of Technology, Dresden, Germany; 8 Department of Psychiatry, University of Toronto, Toronto, Canada; 9 Institute of Medical Science, University of Toronto, Toronto, Canada

**Keywords:** Alcohol consumption, Empirical distribution, Gamma distribution, Log-Normal distribution, Weibull distribution, Population-Attributable Fraction, Exposure distribution, Up-estimation, Per capita consumption, Mean, Standard deviation

## Abstract

**Background:**

The goals of our study are to determine the most appropriate model for alcohol consumption as an exposure for burden of disease, to analyze the effect of the chosen alcohol consumption distribution on the estimation of the alcohol Population- Attributable Fractions (PAFs), and to characterize the chosen alcohol consumption distribution by exploring if there is a global relationship within the distribution.

**Methods:**

To identify the best model, the Log-Normal, Gamma, and Weibull prevalence distributions were examined using data from 41 surveys from Gender, Alcohol and Culture: An International Study (GENACIS) and from the European Comparative Alcohol Study. To assess the effect of these distributions on the estimated alcohol PAFs, we calculated the alcohol PAF for diabetes, breast cancer, and pancreatitis using the three above-named distributions and using the more traditional approach based on categories. The relationship between the mean and the standard deviation from the Gamma distribution was estimated using data from 851 datasets for 66 countries from GENACIS and from the STEPwise approach to Surveillance from the World Health Organization.

**Results:**

The Log-Normal distribution provided a poor fit for the survey data, with Gamma and Weibull distributions providing better fits. Additionally, our analyses showed that there were no marked differences for the alcohol PAF estimates based on the Gamma or Weibull distributions compared to PAFs based on categorical alcohol consumption estimates. The standard deviation of the alcohol distribution was highly dependent on the mean, with a unit increase in alcohol consumption associated with a unit increase in the mean of 1.258 (95% CI: 1.223 to 1.293) (R^2 ^= 0.9207) for women and 1.171 (95% CI: 1.144 to 1.197) (R^2 ^= 0. 9474) for men.

**Conclusions:**

Although the Gamma distribution and the Weibull distribution provided similar results, the Gamma distribution is recommended to model alcohol consumption from population surveys due to its fit, flexibility, and the ease with which it can be modified. The results showed that a large degree of variance of the standard deviation of the alcohol consumption Gamma distribution was explained by the mean alcohol consumption, allowing for alcohol consumption to be modeled through a Gamma distribution using only average consumption.

## Introduction

Alcohol consumption is a component cause [1] for over 200 International Classification of Diseases (ICD-10) three-digit codes [2,3]. In other words, a fraction, usually called the Population-Attributable Fraction (PAF) of the incidence of these diseases, would disappear if exposure to one of the causal components was eliminated [4-7] (in the case of alcohol, under the counterfactual scenario of every person being a lifetime abstainer). The proportion of the diseases caused by alcohol consumption in a component cause model for a population is determined by both the patterns and volume of alcohol consumption and by the relative risks associated with each exposure level [3,8]. For most major diseases where alcohol plays a role (for example, alcohol-attributable cancers, pancreatitis, and cirrhosis of the liver), the average volume of alcohol consumption alone was found to be an adequate predictor of the risk [3,8-10]; however, some diseases and injuries (for example, ischemic heart disease, unintentional injuries, and intentional injuries) were found to be also dependent on drinking patterns [11-14].

The calculation of an alcohol PAF involves a three-stage process: 1) estimation of an exposure distribution of alcohol, 2) establishment of the relative risk function, and 3) the solving of the equation for the PAF [15]. Since the distribution of alcohol consumption on an international level has not been agreed upon, the common approach is to estimate the PAF using categorical measurements rather than modeling it in a more mathematically appropriate continuous manner [16,17]. The mathematical expression is as follows:(Formula 1)

$$PAF=\frac{\overset{k}{\underset{i=1}{\sum }}P_{i}\left(RR_{i}-1\right)}{\overset{k}{\underset{i=1}{\sum }}P_{i}\left(RR_{i}-1\right)+1}$$

where *i *is the exposure category with baseline exposure or no exposure, *i = 0, RR_i _*is the relative risk at exposure level *i *compared to no consumption, and *P_i _*is the prevalence of the *j^th ^*category of exposure.

When a continuous distribution for the volume of alcohol consumption is used, this calculation can be represented by the following formula:(Formula 2)

$$PAF\left(x\right)=\frac{P_{a}RR_{a}\;+P_{ex}RR_{ex}+\;\overset{150}{\underset{0}{\int }}P\left(x\right)RR\left(x\right)dx-1}{P_{a}RR_{a}\;+P_{ex}RR_{ex}+\;\overset{150}{\underset{0}{\int }}P\left(x\right)RR\left(x\right)dx}$$

where *P_a _*is the prevalence of lifetime abstainers, *RR_a _*is the relative risk of lifetime abstainers, *P_ex _*is the prevalence of former drinkers, *RR_ex _*is the relative risk of former drinkers, *x *is the average volume of alcohol consumption per day, *P(x) *is the prevalence of alcohol consumption, and *RR(x) *is the relative risk of drinkers [15]. Although this is the most accurate way to calculate a PAF, it requires that the distribution of alcohol consumption be known. Previous attempts at modeling alcohol consumption using a Log-Normal distribution have been criticized for various reasons [18,19]; however, the Log-Normal distribution has provided adequate approximations for most applications [20,21]. Recently, more adaptable distributions such as the Gamma distribution have been favored over the Log-Normal distribution [15,22], and it has been suggested that a mixing of distributions is needed to separately model the frequency of drinking and the quantity of alcohol consumed [23].

There are two main instruments to monitor alcohol exposure currently used by countries and international organizations: 1) general population surveys and 2) estimates of per capita consumption, where per capita consumption is an aggregate measure of recorded, unrecorded, and tourist per capita consumption of alcohol (derived from sales, production, and other economic statistics) [9,24,25]. These instruments, however, have limitations [26].

There are no available surveys for many countries, and in some cases where they do exist they do not allow for the accurate estimation of the volume of consumption, as these surveys only ask about the absence or presence of drinking [27]. Existing surveys often considerably underestimate real consumption levels [28-30] by typically covering only 30% to 60% of alcohol sales [26]. As a result, per capita consumption figures are considered to be a best estimate of overall volume of consumption in a country [31]; however, per capita consumption does not provide any disaggregated statistic and, thus, does not provide age- and gender-specific consumption estimates. Since in some instances the risk relationship between alcohol consumption and disease-specific mortality is dependent on gender as well as on age, alcohol exposure by gender and age is required to estimate the PAF and to calculate the alcohol-attributable burden of disease in a population [3].

The problems noted above with respect to surveys lead to an underestimated burden of disease attributable to alcohol consumption when PAFs are calculated from population data without adjustment. As a consequence, methods have been developed to triangulate both average alcohol consumption derived from population surveys and from per capita consumption information [15,26]. However, current PAF calculation methods are based on categorical estimates of consumption with alcohol consumption being corrected by multiplying the two top alcohol consumption categories by the inverse of the estimated undercoverage (per capita consumption/the estimated per capita consumption from the survey) [17]. For most categories of disease where there is an association with volume of alcohol consumption, the dose-response relationship is nonlinear and, thus, distribution estimates of alcohol consumption by age and gender are required for accurate estimates of alcohol PAFs [3].

Given the recent recognition of the need to strengthen and disseminate information about alcohol as outlined in the World Health Organization's strategy to reduce harmful consumption of alcohol [32], there is a need to find an appropriate model for exposure, prevalence, and distribution of alcohol consumption that can easily be modeled to make the fit more compatible with per capita consumption data and that also has properties that make it possible to estimate the exposure distribution for countries that lack survey data except for estimates of prevalence of abstention. Thus, the first aim of this study is to assess internationally if alcohol consistently follows one of the three well-known right-skewed distributions, Log-Normal, Gamma, or Weibull, and to determine if the chosen exposure distribution has a significant effect on the estimation of a PAF, using the PAFs for pancreatitis, diabetes, and breast cancer as examples. The second aim of this study is to investigate if a global relationship between parameters exists so that a distribution of alcohol consumption can be estimated based on mean alcohol consumption.

## Methods

### Description of underlying surveys

This study used data from Gender, Alcohol and Culture: An International Study (GENACIS), from the European Comparative Alcohol Study (ECAS), and from the STEPwise approach to Surveillance (STEPS). Survey data were collected for the average volume of consumption for Argentina, Australia (two surveys from Australia were used: Australia and Australia1), Austria, Belize, Brazil, Canada, Costa Rica, Czech Republic, Denmark, Finland, France, Germany, Hungary, Iceland, India, Ireland, Isle of Man, Israel, Italy, Japan, Kazakhstan, Mexico, Netherlands, Nicaragua, Nigeria, Norway, Peru, Spain, Sri Lanka, Sweden, Switzerland, Uganda, United Kingdom, Uruguay, and the United States of America from GENACIS (three surveys from the United States of America were used: USA1, USA2, and USA3; USA1 was a 2001 longitudinal study that surveyed women only, and USA2 and USA3 were 1995-1996 and 2000 National Alcohol Surveys, respectively); for Finland, France, Germany, Italy, Sweden, and the United Kingdom from ECAS; and for Cameroon, Côte D' Ivoire, Dominica, Democratic Republic of the Congo, Eritrea, Kuwait, Mali, Mozambique, American Samoa, Barbados, Benin, Botswana, Cape Verde, Republic of the Congo, Cook Islands, Indonesia, Madagascar, St. Kitts and Nevis, Swaziland, Zambia, Fiji, Kiribati, Marshall Islands, Mongolia, Nauru, Solomon Islands, Tokelau, Tonga, Vanuatu, Micronesia, and Samoa from STEPS. (For information on sampling methodology and the questions used in GENACIS surveys see [33-35], ECAS see [30], and STEPS see [36]). For most of the GENACIS surveys and for the ECAS surveys alcohol consumption was measured by a beverage-specific usual quantity-frequency technique (i.e., asking separate questions on usual frequency of drinking, and then eliciting the usual quantity per drinking occasion), and in the remaining GENACIS surveys alcohol consumption was measured by a global quantity-frequency measure. In the STEPS surveys alcohol consumption was measured in standard drinks consumed in the seven days preceding the survey.

All data from surveys were divided by sex and age into eight age groups; 15-24, 25-34, 35-44, 45-54, 55-64, 65-74, 75-84, and 85 +.

### Methods for fitting the distributions

As alcohol consumption distributions have been shown to have a unimodal shape, [19,37,38] we evaluated the fit of the Log-Normal, Gamma, and Weibull distributions (unimodal distributions commonly used to fit right-skewed empirical data) to determine the most appropriate distribution to model alcohol consumption from national survey data. The Log-Normal, Gamma, and Weibull probability densities are similar in shape, but have significantly different tail behaviors. In the past, alcohol consumption has been more commonly modeled by the Log-Normal distribution as it is used to model continuous random quantities that are right-skewed and is based on the normal distribution, making it easy to fit, test, and modify [20,21]. Although alcohol consumption is frequently modeled using the Log-Normal distribution, empirical distributions often deviate considerably from the Log-Normal model. In comparison, the Gamma and Weibull distributions have a scale parameter and a shape parameter, making them more adaptable since the scale parameter can stretch or compress the distribution.

The Log-Normal distribution is a function of the mean (μ) and standard deviation (σ) parameters, and describes a random variable x where log (x) is normally distributed. The probability density function of the Log-Normal distribution can be expressed as follows:

$$f\left(x;\mu ,\sigma \right)=\frac{1}{x\sigma \sqrt{2\pi }}\exp\left\{-\frac{{\left(\log\;x-\mu \right)}^{2}}{2\sigma^{2}}\right\}$$

where x > 0 and -∞ < μ < ∞, σ > 0 The Gamma distribution is characterized by a shape (κ) and a scale parameter (θ), has a mean of κθ and a standard deviation of $\sqrt{\kappa \theta^{2}}.$ The probability density function of the Gamma distribution can be expressed as follows:

$$f\left(x;\kappa ,\theta \right)=\frac{x^{\kappa -1}}{\theta^{\kappa }\Gamma \left(\kappa \right)}\exp\left\{-\frac{x}{\theta }\right\}$$

where x > 0, κ > 0, θ > 0 and $\Gamma \left(\kappa \right)=\overset{\infty }{\underset{0}{\int }}t^{\kappa -1}\exp\left\{-t\right\}dt$ Similar to the Gamma distribution, the Weibull distribution is commonly characterized by a shape (γ) and a scale parameter (θ). The Weibull distribution has a mean of $\theta \;\Gamma \left(\frac{1}{\gamma }+1\right)$ and a standard deviation of $\theta \;\sqrt{\Gamma \left(\frac{2}{\gamma }+1\right)-\Gamma {\left(\frac{1}{\gamma }+1\right)}^{2}}$, where $\Gamma \left(x\right)=\overset{\infty }{\underset{0}{\int }}t^{x-1}\exp\left\{-t\right\}dt$ is the Gamma function evaluated at *x*. The probability density function of the Weibull distribution is expressed as follows:

$$f\left(x;\theta ,\gamma \right)=\frac{\gamma }{\theta }{\left(\frac{x}{\theta }\right)}^{\gamma -1}\exp\left\{-{\left(\frac{x}{\theta }\right)}^{\gamma }\right\}$$

where x ≥ 0, γ > 0, θ > 0 Maximum likelihood estimation was used to fit all three distribution models to the drinking population data obtained from GENACIS and ECAS. All missing values were excluded from the fitted models. The Newton-Raphson algorithm was used to optimize the likelihood equations solving for the maximum likelihood estimates of the unknown parameters [39]. Data values of alcohol consumption over 300 g/day were truncated to 300 g/day. Numerical integration utilizing the trapezoidal rule was used to characterize each distribution.

### Method for deriving the alcohol PAF

We performed a sensitivity analysis where the alcohol PAFs for pancreatitis, diabetes, and breast cancer were calculated using a continuous model (Log-Normal, Gamma, and Weibull) and using a categorical model in order to see if the chosen exposure distribution had an effect on the estimation of the alcohol PAF. All PAFs were calculated with zero alcohol consumption as the counterfactual scenario, similarly to the Comparative Risk Analysis for alcohol. This counterfactual scenario under certain circumstances of a light drinking average alcohol consumption without heavy drinking occasions may not reflect the theoretical minimum risk depending on the distribution of diseases and cause of death in a society. However, for this paper these considerations are not relevant. The relative risks of lifetime abstainers and former drinkers for pancreatitis, diabetes, and breast cancer were obtained from the meta-analysis [40-42].

In order to illustrate that the alcohol PAF estimates based on the Gamma distribution model deviated only slightly from the PAF derived from the categorical model, we calculated the difference between the PAFs calculated for both models.

### Methods for characterizing the gamma distributions

The Gamma distribution can be characterized by a shape (κ) and a scale parameter (θ), where the mean and the standard deviation of the Gamma distribution can be obtained directly from the parameter estimates as follows:

$$\mu =\kappa \theta \;and\;\sigma =\sqrt{\kappa \theta^{2}}$$

Since the mean of the Gamma distribution is equal to the mean of the empirical distribution, the mean of the Gamma distribution does not need to be estimated from the shape and scale parameters.

A maximum likelihood algorithm (see description above) was used to obtain the shape and scale parameters using the maximum likelihood function for the shape and scale parameters of the Gamma distribution:

$$l\left(\kappa ,\theta \right)=\left(\kappa -1\right)\overset{N}{\underset{i=1}{\sum }}\ln\left(x_{i}\right)-\overset{N}{\underset{i=1}{\sum }}\frac{x_{i}}{\theta }-N\cdot \kappa \cdot \ln\left(\theta \right)-N\cdot \ln\left(\Gamma \left(\kappa \right)\right)$$

#### Regression analysis

The maximum likelihood method was used to fit a Gamma model in order to summarize the alcohol consumption of 66 countries by gender and age (in total 851 datasets [422 for women; 429 for men]). After the data was fit by a Gamma model, the relationship between the Gamma mean and the Gamma standard deviation was examined using various general linear models. The performance of the general linear models was then assessed by how well the assumption of homoscedasticity was upheld and based on the distribution of the residuals.

All data analyses were performed in R version 2.13.0 [43].

## Results

### Modeling alcohol consumption as a distribution

The three distributions, Log-Normal, Gamma, and Weibull, were fit to 41 datasets; parameter estimates are outlined in Table 1 for women and in Table 2 for men. The mean and standard deviation estimates from the empirical data and the estimates from each fitted model are summarized in Table 3 for women and in Table 4 for men. When comparing the empirical mean to each distribution's mean, we observed that the mean estimates from the Weibull distribution were much closer to the empirical mean than were the Log-Normal distribution mean estimates, while the mean estimates from the Gamma distribution were equal to the empirical mean. When comparing the standard deviation estimates, the estimates from the Log-Normal distribution deviated furthest from the empirical data, while there was no statistically significant difference between the empirical standard deviation estimate and the standard deviation estimates from either of the Weibull or the Gamma distributions.

**Table 1:** Parameter estimates from Log-Normal, Gamma, and Weibull models for women from 43 datasets

	Log-Normal model parameter estimates		Gamma model parameter estimates		Weibull model parameter estimates	
**Country**	**Mean**	**Standard deviation**	**Scale**	**Shape**	**Scale**	**Shape**

Argentina	0.14	1.93	9.17	0.48	2.92	0.60
Australia	0.57	1.88	11.75	0.51	4.33	0.64
Australia 1	0.47	1.57	8.57	0.56	3.55	0.67
Austria	1.91	0.92	8.45	1.26	10.85	1.05
Belize	0.64	1.51	13.44	0.50	4.17	0.62
Brazil	1.09	2.10	36.30	0.41	8.18	0.54
Canada	1.06	1.41	9.92	0.69	5.78	0.77
Costa Rica	-0.28	1.81	7.20	0.45	1.88	0.57
Czech Republic	1.04	1.70	16.47	0.54	6.49	0.66
Denmark	1.37	1.40	9.37	0.84	7.46	0.89
ECAS: Finland	1.07	1.20	6.51	0.88	5.26	0.87
ECAS: France	0.94	1.56	10.94	0.63	5.51	0.72
ECAS: Germany	1.05	1.34	9.21	0.72	5.53	0.78
ECAS: Italy	1.37	1.59	15.91	0.64	8.45	0.74
ECAS: Sweden	0.90	1.17	4.23	1.02	4.30	0.99
ECAS: UK	1.70	1.48	19.03	0.69	11.13	0.77
Finland	0.47	1.67	7.08	0.61	3.47	0.72
France	1.62	1.05	9.30	0.98	8.75	0.92
Germany	1.30	1.47	12.42	0.70	7.43	0.78
Hungary	-0.82	1.89	4.36	0.44	1.11	0.58
Iceland	0.82	1.31	5.78	0.81	4.23	0.84
India	1.31	2.16	42.29	0.42	10.39	0.55
Ireland	2.01	1.23	15.55	0.91	13.53	0.91
Isle of Man	1.18	1.85	16.98	0.57	7.59	0.69
Israel	-0.05	1.98	12.55	0.40	2.52	0.54
Italy	1.52	1.39	12.97	0.77	8.95	0.83
Japan	-0.15	2.18	14.32	0.37	2.53	0.50
Kazakhstan	-0.52	1.93	6.67	0.42	1.52	0.56
Mexico	-1.15	1.63	5.03	0.37	0.76	0.53
Netherlands	1.44	1.11	8.33	0.94	7.43	0.91
Nicaragua	0.91	1.49	26.83	0.43	5.54	0.57
Nigeria	1.84	2.31	65.85	0.43	18.29	0.56
Norway	0.61	1.58	7.07	0.66	3.85	0.75
Peru	0.16	0.91	1.62	1.18	1.89	0.98
Spain	1.07	1.78	13.31	0.61	6.58	0.72
Sri Lanka	-2.28	1.69	3.31	0.30	0.27	0.46
Sweden	0.44	1.26	4.15	0.79	2.93	0.83
Switzerland	1.39	1.25	8.07	0.93	7.21	0.93
Uganda	0.98	2.09	34.50	0.40	7.39	0.53
Uruguay	0.19	1.90	11.60	0.45	3.10	0.58
USA 1	0.18	1.96	12.42	0.43	3.16	0.56
USA 2	0.30	1.62	11.49	0.47	3.12	0.59
USA 3	0.23	1.67	9.85	0.48	2.94	0.61

**Table 2:** Parameter estimates from Log-Normal, Gamma, and Weibull models for men from 41 datasets

	Log-Normal model parameter estimates		Gamma model parameter estimates		Weibull model parameter estimates	
**Country**	**Mean**	**Standard deviation**	**Scale**	**Shape**	**Scale**	**Shape**

Argentina	1.84	1.68	25.33	0.64	13.62	0.75
Australia	1.63	1.69	18.79	0.67	10.99	0.78
Austria	2.85	0.96	19.72	1.33	27.52	1.13
Belize	2.06	1.55	37.69	0.59	16.85	0.69
Brazil	1.57	2.01	47.07	0.44	12.55	0.57
Canada	1.96	1.42	21.64	0.74	14.04	0.81
Costa Rica	1.13	1.87	23.50	0.49	7.71	0.61
Czech Republic	2.58	1.55	38.84	0.75	26.59	0.83
Denmark	2.28	1.24	18.05	0.98	17.33	0.96
ECAS: Finland	2.22	1.18	16.68	0.99	16.13	0.95
ECAS: France	2.18	1.48	26.19	0.75	17.56	0.82
ECAS: Germany	1.92	1.33	16.43	0.84	12.84	0.87
ECAS: Italy	2.22	1.40	20.68	0.87	17.43	0.92
ECAS: Sweden	1.79	1.26	13.48	0.87	10.94	0.88
ECAS: UK	2.85	1.30	38.19	0.88	31.78	0.90
Finland	1.76	1.51	17.08	0.75	11.58	0.83
France	2.44	1.25	25.29	0.88	21.08	0.90
Germany	2.27	1.37	21.29	0.88	18.07	0.92
Hungary	1.10	1.81	17.13	0.55	6.95	0.67
Iceland	1.64	1.25	9.84	0.96	9.17	0.95
India	2.24	1.95	69.20	0.49	23.75	0.62
Ireland	3.04	1.18	38.57	0.98	36.94	0.95
Isle of Man	2.22	1.78	39.38	0.63	20.51	0.74
Israel	1.02	1.87	22.11	0.48	6.85	0.61
Italy	2.44	1.30	21.80	0.96	20.92	0.99
Japan	1.63	2.19	37.45	0.49	13.60	0.63
Kazakhstan	1.87	1.76	36.80	0.55	14.69	0.67
Mexico	1.34	1.90	33.23	0.46	9.68	0.59
Netherlands	2.28	1.17	17.45	1.00	17.27	0.98
Nicaragua	2.03	1.52	38.43	0.58	16.28	0.68
Nigeria	2.47	1.78	55.90	0.60	26.97	0.71
Norway	1.66	1.44	15.92	0.74	10.25	0.80
Peru	1.13	1.17	8.89	0.76	5.60	0.79
Spain	2.28	1.49	25.30	0.81	19.04	0.87
Sri Lanka	1.30	2.18	57.93	0.37	10.71	0.51
Sweden	1.12	1.32	8.20	0.79	5.83	0.83
Switzerland	2.37	1.12	17.65	1.05	18.27	0.97
Uganda	2.75	1.79	70.07	0.61	35.42	0.73
Uruguay	1.69	1.84	34.78	0.52	12.88	0.64
USA 2	1.41	1.72	25.65	0.53	9.50	0.64
USA 3	1.32	1.80	28.58	0.49	9.04	0.61

**Table 3:** Mean and standard deviation estimates from the empirical data, Log-Normal model, Gamma model, and the Weibull model for alcohol consumption of women from 43 datasets

	Empirical data			Log-Normal model		Gamma model		Weibull model	
**Country**	**Count**	**Mean**	**Standard deviation**	**Mean**	**Standard deviation**	**Mean**	**Standard deviation**	**Mean**	**Standard deviation**

Argentina	381	4.38	6.77	7.35	46.50	4.38	6.34	4.39	7.69

Australia	1172	6.04	9.52	10.40	60.39	6.04	8.42	6.06	9.90

Australia 1	3002	4.84	7.81	5.47	17.86	4.84	6.44	4.69	7.22

Austria	1916	10.62	13.26	10.36	11.94	10.62	9.47	10.66	10.20

Belize	386	6.74	16.63	5.92	17.44	6.74	9.52	5.98	10.02

Brazil	283	14.80	29.63	26.75	240.21	14.80	23.18	14.27	28.60

Canada	5850	6.88	10.79	7.82	19.76	6.88	8.26	6.75	8.90

Costa Rica	367	3.21	6.33	3.90	19.86	3.21	4.81	3.00	5.57

Czech Republic	1023	8.97	15.12	12.08	50.02	8.97	12.16	8.74	13.71

Denmark	1042	7.89	8.85	10.48	26.03	7.89	8.59	7.89	8.85

ECAS: Finland	469	5.71	9.65	6.00	10.77	5.71	6.09	5.63	6.47

ECAS: France	382	6.85	9.83	8.64	27.71	6.85	8.66	6.77	9.54

ECAS: Germany	512	6.93	21.77	7.05	15.85	6.62	7.80	6.39	8.30

ECAS: Italy	404	10.23	14.99	14.08	48.17	10.23	12.76	10.17	13.94

ECAS: Sweden	433	4.32	4.58	4.87	8.35	4.32	4.28	4.32	4.36

ECAS: UK	498	13.14	19.31	16.34	46.06	13.14	15.81	12.97	17.02

Finland	882	4.35	7.83	6.45	25.27	4.35	5.55	4.28	6.07

France	4206	9.14	11.79	8.78	12.42	9.14	9.22	9.08	9.83

Germany	4164	8.72	12.97	10.88	30.24	8.72	10.41	8.60	11.17

Hungary	883	1.92	5.31	2.62	15.31	1.92	2.90	1.75	3.22

Iceland	1072	4.70	7.41	5.34	11.37	4.70	5.21	4.63	5.53

India	85	17.67	26.94	38.01	388.74	17.67	27.33	17.88	35.44

Ireland	378	14.20	17.69	16.05	30.35	14.20	14.86	14.14	15.54

Isle of Man	469	9.67	13.20	17.91	97.33	9.67	12.81	9.77	14.57

Israel	1938	4.98	12.52	6.70	46.91	4.98	7.91	4.46	9.04

Italy	1219	9.93	11.72	11.91	28.76	9.93	11.35	9.90	12.01

Japan	864	5.27	11.72	9.17	97.39	5.27	8.68	5.02	11.15

Kazakhstan	401	2.80	7.91	3.78	23.87	2.80	4.32	2.50	4.78

Mexico	1406	1.88	7.32	1.20	4.39	1.88	3.07	1.37	2.82

Netherlands	1505	7.84	10.50	7.83	12.20	7.84	8.08	7.78	8.58

Nicaragua	147	11.43	34.88	7.52	21.56	11.43	17.51	8.94	16.78

Nigeria	200	28.45	41.91	91.55	1322.58	28.45	43.28	30.12	57.50

Norway	1004	4.64	7.03	6.39	21.38	4.64	5.73	4.59	6.21

Peru	620	1.91	3.07	1.78	2.03	1.91	1.76	1.90	1.95

Spain	427	8.07	11.17	14.34	69.00	8.07	10.36	8.12	11.50

Sri Lanka	38	1.00	2.93	0.42	1.70	1.00	1.82	0.64	1.63

Sweden	2226	3.29	4.51	3.42	6.75	3.29	3.69	3.24	3.94

Switzerland	5362	7.50	10.07	8.77	17.04	7.50	7.78	7.48	8.09

Uganda	280	13.78	26.60	23.46	206.14	13.78	21.80	13.25	27.17

Uruguay	375	5.17	12.02	7.35	43.87	5.17	7.75	4.91	9.05

USA 1	854	5.37	10.39	8.11	54.33	5.37	8.17	5.21	9.95

USA 2	1310	5.35	14.44	5.00	17.90	5.35	7.84	4.76	8.46

USA 3	2274	4.75	10.65	5.09	19.93	4.75	6.84	4.36	7.57

**Table 4:** Mean and standard deviation estimates from the empirical data, Log-Normal model, Gamma model, and the Weibull model for alcohol consumption of men from 41 datasets

	Empirical data			Log-Normal model		Gamma model		Weibull model	
**Country**	**Count**	**Mean**	**Standard deviation**	**Mean**	**Standard deviation**	**Mean**	**Standard deviation**	**Mean**	**Standard deviation**

Argentina	359	16.26	21.80	25.79	102.88	16.26	20.29	16.29	22.18

Australia	882	12.63	15.09	21.31	86.21	12.63	15.40	12.73	16.58

Austria	2697	26.23	26.25	27.23	33.35	26.23	22.75	26.35	23.43

Belize	957	22.52	41.05	26.19	83.46	22.31	29.00	21.51	31.79

Brazil	325	20.78	37.89	35.81	265.46	20.78	31.27	20.16	37.64

Canada	4833	16.09	24.60	19.65	50.51	16.02	18.62	15.84	19.82

Costa Rica	285	11.47	18.97	17.91	101.55	11.47	16.42	11.30	19.36

Czech Republic	1121	29.19	32.98	43.56	137.44	29.19	33.67	29.27	35.28

Denmark	865	17.68	21.18	21.03	39.93	17.68	17.87	17.66	18.42

ECAS: Finland	462	16.53	21.56	18.48	32.09	16.53	16.60	16.49	17.32

ECAS: France	415	19.75	28.51	26.44	74.41	19.66	22.69	19.55	23.98

ECAS: Germany	328	13.81	19.83	16.65	36.89	13.81	15.06	13.73	15.76

ECAS: Italy	434	18.06	19.09	24.71	61.39	18.06	19.33	18.08	19.57

ECAS: Sweden	449	11.77	17.59	13.28	26.13	11.77	12.60	11.66	13.29

ECAS: UK	361	34.95	54.61	40.33	85.07	33.62	35.83	33.48	37.34

Finland	864	12.88	17.32	18.14	53.44	12.88	14.83	12.84	15.63

France	4697	22.24	25.21	24.83	47.92	22.24	23.72	22.18	24.67

Germany	3510	18.79	20.74	24.77	58.38	18.79	20.00	18.79	20.45

Hungary	991	9.38	15.16	15.50	78.87	9.38	12.68	9.25	14.34

Iceland	1013	9.42	11.04	11.18	21.62	9.42	9.63	9.40	9.94

India	498	34.82	54.78	63.16	417.87	34.20	48.65	34.40	58.26

Ireland	385	37.82	43.73	42.23	73.86	37.82	38.19	37.74	39.61

Isle of Man	420	24.90	36.39	45.31	217.60	24.64	31.15	24.77	34.16

Israel	2005	10.59	19.71	15.91	89.50	10.59	15.30	10.19	17.71

Italy	1429	20.98	19.35	26.89	56.90	20.98	21.39	20.98	21.13

Japan	1009	18.51	25.29	55.72	605.09	18.51	26.33	19.42	32.43

Kazakhstan	401	20.55	40.31	30.49	139.45	20.27	27.31	19.50	30.13

Mexico	1833	15.48	30.37	23.46	141.72	15.37	22.60	14.86	26.61

Netherlands	1679	17.47	18.78	19.46	33.31	17.47	17.46	17.46	17.89

Nicaragua	263	22.26	40.29	24.27	73.44	22.26	29.25	21.16	31.91

Nigeria	439	33.63	45.76	58.17	279.62	33.38	43.19	33.66	48.39

Norway	945	11.78	19.42	14.67	38.42	11.78	13.70	11.59	14.57

Peru	425	6.76	15.68	6.10	10.43	6.76	7.75	6.42	8.24

Spain	603	20.44	24.47	29.64	84.47	20.44	22.74	20.43	23.56

Sri Lanka	323	21.87	43.99	39.56	426.76	21.65	35.42	20.87	45.74

Sweden	2348	6.49	9.17	7.30	15.79	6.49	7.30	6.42	7.74

Switzerland	5126	18.55	24.86	20.15	32.01	18.54	18.09	18.50	19.05

Uganda	378	42.93	52.50	78.02	382.06	42.80	54.76	43.42	61.01

Uruguay	305	18.32	34.55	29.22	154.64	18.16	25.14	17.75	28.56

USA 2	1499	13.51	24.00	17.81	75.66	13.51	18.62	13.12	21.16

USA 3	2300	14.18	32.43	19.12	95.29	13.93	19.95	13.20	22.54

**Figure 1:**
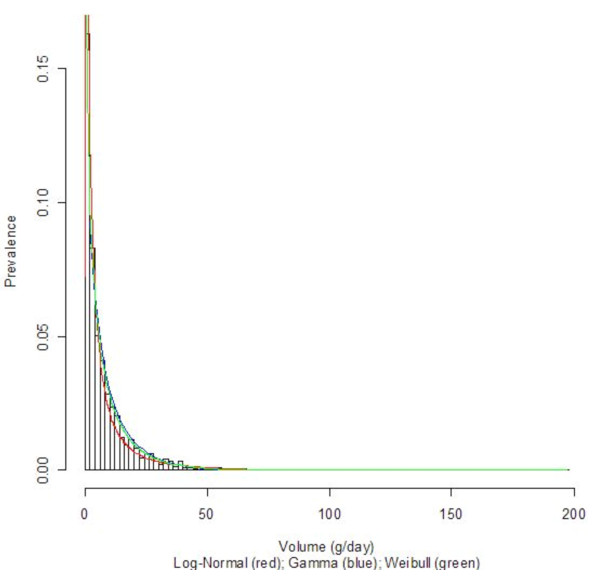
**Alcohol consumption distribution in grams per day of pure alcohol for women in Germany**. Alcohol consumption distribution in grams per day of pure alcohol for women in Germany.

**Figure 2:**
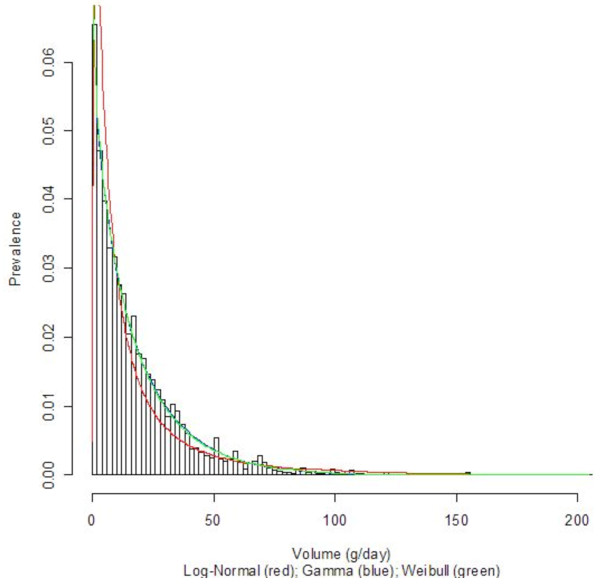
**Alcohol consumption distribution in grams per day of pure alcohol for men in Germany**.

**Figure 3:**
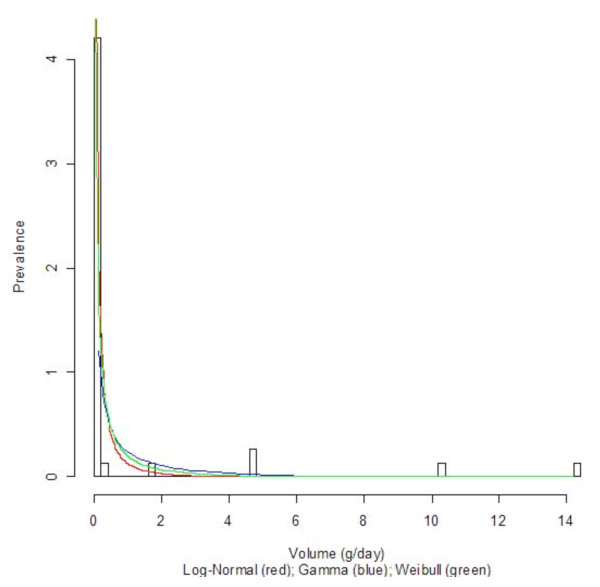
**Alcohol consumption distribution in grams per day of pure alcohol for women in Sri Lanka**.

**Figure 4:**
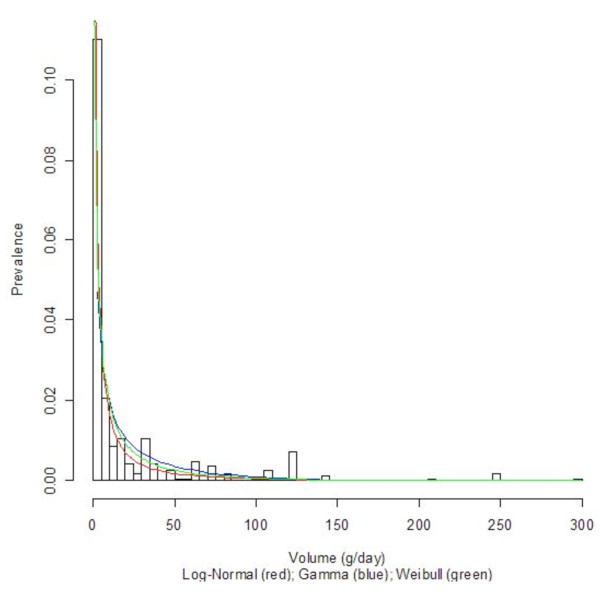
**Alcohol consumption distribution in grams per day of pure alcohol for men in Sri Lanka**. Alcohol consumption distribution in grams per day of pure alcohol for men in Sri Lanka.

**Figure 5:**
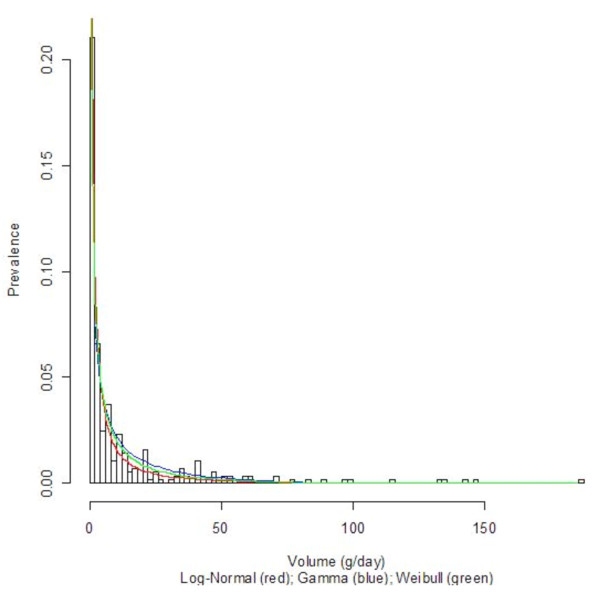
**Alcohol consumption distribution in grams per day of pure alcohol for women in Uganda**.

**Figure 6:**
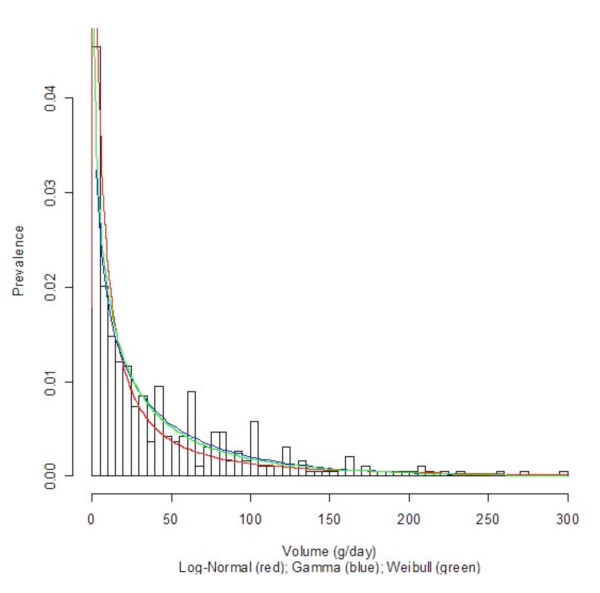
**Alcohol consumption distribution in grams per day of pure alcohol for men in Uganda**.

Three countries with diverse economic conditions and drinking patterns, namely Germany, Sri Lanka, and Uganda, were selected to display their density curves (Log-Normal, Gamma, and Weibull) superimposed on the population-based data histograms; see Figures 1, 2, 3, 4, 5, and 6 for both women and men. We observed a common trend among men in Figures 2, 4, and 6: the Log-Normal distribution tended to underestimate the number of men who drank 25 g/day to 50 g/day, whereas the Gamma and Weibull distributions accurately estimated alcohol consumption for these populations. A similar trend was observed with respect to women from Germany and Uganda who drank between 10 g/day to 30 g/day and for Sri Lankan women who drank between 0.5 g/day to 2.0 g/day.

Alcohol PAF estimates modeled using the Log-Normal, Gamma, and Weibull distributions, together with the proportion estimates for lifetime abstainers and former drinkers, are listed in Table 5 for breast cancer (women), Tables 6 and 7 for diabetes (women and men, respectively), and Tables 8 and 9 for pancreatitis (women and men, respectively).

**Table 5:** Proportion estimates for lifetime abstainers and former drinkers, as well as Population-Attributable Fraction (PAF) estimates for breast cancer using a categorical model and continuous models (Gamma, Log-Normal, and Weibull) for women

	Proportions		PAF estimates				
**Country**	**Abstainers**	**Former drinkers**	**Categorical**	**Gamma model**	**Log-Normal model**	**Weibull model**	***PAF*_categorical - _*PAF*_gamma_**

Argentina	0.06355	0.29933	0.14923	0.1354	0.15384	0.1362	0.01383
Australia	0.04951	0.13319	0.10734	0.09444	0.12996	0.096	0.0129
Australia 1	0.12341	0.06414	0.08152	0.06024	0.07021	0.05996	0.02128
Austria	0.17941	0.3288	0.16652	0.16302	0.1632	0.16338	0.0035
Belize	0.59903	0.21449	0.10145	0.09595	0.09592	0.0951	0.0055
Brazil	0.22741	0.36006	0.19526	0.18374	0.20113	0.18618	0.01152
Canada	0.09532	0.16116	0.1189	0.10649	0.11779	0.10619	0.01241
Costa Rica	0.19253	0.37923	0.16137	0.15162	0.15593	0.15135	0.00975
Czech Republic	0.05538	0.14665	0.13325	0.11822	0.14643	0.11888	0.01503
Denmark	0.00971	0.07061	0.10045	0.09091	0.12153	0.0911	0.00954
ECAS: Finland	0.0748	0.00197	0.06369	0.0474	0.05292	0.04699	0.01629
ECAS: France	0.27238	0	0.05939	0.04492	0.06499	0.04519	0.01447
ECAS: Germany	0.18471	0	0.07463	0.04827	0.05679	0.0471	0.02636
ECAS: Italy	0.21206	0.00195	0.08903	0.07422	0.11217	0.07535	0.01481
ECAS: Sweden	0.132	0.002	0.04803	0.03386	0.03994	0.03388	0.01417
ECAS: UK	0.14286	0	0.11594	0.10312	0.13957	0.10394	0.01282
Finland	0.06491	0.04057	0.06973	0.05056	0.07463	0.05056	0.01917
France	0.03552	0.41525	0.19287	0.18747	0.18762	0.18745	0.0054
Germany	0.02588	0.03691	0.10094	0.08681	0.11568	0.08677	0.01413
Hungary	0.17718	0.05964	0.06264	0.03701	0.0453	0.0363	0.02563
Iceland	0.07396	0.08261	0.08375	0.06784	0.07546	0.0675	0.01591
India	0.84014	0.10204	0.05502	0.05365	0.05764	0.05469	0.00137
Ireland	0.23933	0.05937	0.1181	0.11259	0.1345	0.11288	0.00551
Isle of Man	0.01838	0.11949	0.12723	0.1185	0.1757	0.12157	0.00873
Israel	0.42882	0	0.04408	0.02458	0.0391	0.02325	0.0195
Italy	0.19622	0.06094	0.10517	0.0899	0.11227	0.09029	0.01527
Japan	0.15058	0.08415	0.0886	0.06775	0.09511	0.06877	0.02085
Kazakhstan	0.10143	0.26307	0.13401	0.11568	0.12401	0.11479	0.01833
Mexico	0.40553	0.17212	0.09092	0.07588	0.07463	0.07449	0.01504
Netherlands	0.14467	0.17495	0.12055	0.11305	0.11531	0.11294	0.0075
Nicaragua	0.50282	0.39336	0.16442	0.15622	0.15366	0.15459	0.0082
Nigeria	0.56034	0.2298	0.16004	0.15402	0.16134	0.15696	0.00602
Norway	0.04049	0.0757	0.08266	0.0662	0.08621	0.06621	0.01646
Peru	0.08966	0.29951	0.13924	0.1245	0.12399	0.12449	0.01474
Spain	0.22908	0.32427	0.1547	0.15056	0.17495	0.15131	0.00414
Sri Lanka	0.8661	0.06949	0.03264	0.03012	0.02992	0.02995	0.00252
Sweden	0.09666	0.1123	0.08539	0.06797	0.06996	0.06777	0.01742
Switzerland	0.19806	0.06082	0.08298	0.07341	0.08702	0.0734	0.00957
Uganda	0.36412	0.26649	0.15735	0.14658	0.1619	0.14889	0.01077
Uruguay	0.17308	0.22596	0.12907	0.11302	0.12813	0.11289	0.01605
USA 1	0.10302	0.13854	0.10544	0.089	0.11203	0.08978	0.01644
USA 2	0.3263	0.18852	0.11255	0.09623	0.09806	0.09469	0.01632
USA 3	0.38019	0.05805	0.06399	0.04719	0.05318	0.04608	0.0168

**Table 6:** Proportion estimates for lifetime abstainers and former drinkers, as well as Population-Attributable Fraction (PAF) estimates for diabetes using a categorical model and continuous models (Gamma, Log-Normal, and Weibull) for women

	Proportions		PAF estimates				
**Country**	**Abstainers**	**Former drinkers**	**Categorical**	**Gamma model**	**Log-Normal model**	**Weibull model**	***PAF*_categorical - _*PAF*_gamma_**

Argentina	0.06355	0.29933	-0.14692	-0.06787	-0.05274	-0.06333	-0.07905
Australia	0.04951	0.13319	-0.24721	-0.15762	-0.12626	-0.15022	-0.08959
Australia 1	0.12341	0.06414	-0.2699	-0.16237	-0.14117	-0.15421	-0.10753
Austria	0.17941	0.3288	-0.10003	-0.09967	-0.09292	-0.09467	-0.00036
Belize	0.59903	0.21449	-0.01794	-0.00596	-0.00215	-0.0034	-0.01198
Brazil	0.22741	0.36006	-0.05408	-0.02384	-0.01732	-0.02246	-0.03024
Canada	0.09532	0.16116	-0.21615	-0.16259	-0.13959	-0.15557	-0.05356
Costa Rica	0.19253	0.37923	-0.06097	-0.00801	-0.00214	-0.00435	-0.05296
Czech Republic	0.05538	0.14665	-0.23423	-0.17322	-0.14215	-0.16336	-0.06101
Denmark	0.00971	0.07061	-0.33046	-0.26493	-0.22239	-0.26241	-0.06553
ECAS: Finland	0.0748	0.00197	-0.32158	-0.25101	-0.22964	-0.24296	-0.07057
ECAS: France	0.27238	0	-0.25029	-0.18164	-0.15523	-0.17478	-0.06865
ECAS: Germany	0.18471	0	-0.2797	-0.21639	-0.19186	-0.20633	-0.06331
ECAS: Italy	0.21206	0.00195	-0.2856	-0.21937	-0.1826	-0.21217	-0.06623
ECAS: Sweden	0.132	0.002	-0.29211	-0.20986	-0.19889	-0.20854	-0.08225
ECAS: UK	0.14286	0	-0.30489	-0.25529	-0.22162	-0.24719	-0.0496
Finland	0.06491	0.04057	-0.29456	-0.18499	-0.16109	-0.18021	-0.10957
France	0.03552	0.41525	-0.10183	-0.08985	-0.08062	-0.08479	-0.01198
Germany	0.02588	0.03691	-0.33041	-0.26864	-0.22619	-0.25848	-0.06177
Hungary	0.17718	0.05964	-0.22547	-0.08457	-0.08059	-0.07914	-0.1409
Iceland	0.07396	0.08261	-0.26082	-0.18598	-0.16929	-0.18056	-0.07484
India	0.84014	0.10204	0.0037	0.00412	0.00483	0.00417	-0.00042
Ireland	0.23933	0.05937	-0.21416	-0.21209	-0.18943	-0.20603	-0.00207
Isle of Man	0.01838	0.11949	-0.27038	-0.20513	-0.15778	-0.19787	-0.06525
Israel	0.42882	0	-0.17003	-0.09966	-0.08491	-0.09059	-0.07037
Italy	0.19622	0.06094	-0.25978	-0.2077	-0.17787	-0.20246	-0.05208
Japan	0.15058	0.08415	-0.23347	-0.11957	-0.09699	-0.10624	-0.1139
Kazakhstan	0.10143	0.26307	-0.14039	-0.04881	-0.04103	-0.0426	-0.09158
Mexico	0.40553	0.17212	-0.08857	-0.02026	-0.01479	-0.01363	-0.06831
Netherlands	0.14467	0.17495	-0.18932	-0.16501	-0.15025	-0.15897	-0.02431
Nicaragua	0.50282	0.39336	0.02923	0.03438	0.03458	0.03517	-0.00515
Nigeria	0.56034	0.2298	-0.00892	0.00013	-0.00065	-0.00187	-0.00905
Norway	0.04049	0.0757	-0.28376	-0.18535	-0.16146	-0.18067	-0.09841
Peru	0.08966	0.29951	-0.12528	-0.04802	-0.04493	-0.04558	-0.07726
Spain	0.22908	0.32427	-0.07944	-0.05418	-0.03553	-0.05184	-0.02526
Sri Lanka	0.8661	0.06949	-0.00659	0.00516	0.00598	0.00625	-0.01175
Sweden	0.09666	0.1123	-0.23299	-0.13662	-0.12713	-0.13302	-0.09637
Switzerland	0.19806	0.06082	-0.24288	-0.20449	-0.18102	-0.20067	-0.03839
Uganda	0.36412	0.26649	-0.05139	-0.02926	-0.02312	-0.02722	-0.02213
Uruguay	0.17308	0.22596	-0.14518	-0.07583	-0.06006	-0.06876	-0.06935
USA 1	0.10302	0.13854	-0.22157	-0.12244	-0.1	-0.11227	-0.09913
USA 2	0.3263	0.18852	-0.11105	-0.06216	-0.05102	-0.05505	-0.04889
USA 3	0.38019	0.05805	-0.16022	-0.09638	-0.08313	-0.08908	-0.06384

**Table 7:** Proportion estimates for lifetime abstainers and former drinkers, as well as Population-Attributable Fraction (PAF) estimates for diabetes using a categorical model and continuous models (Gamma, Log-Normal, and Weibull) for men

	Proportions		PAF estimates				
**Country**	**Abstainers**	**Former drinkers**	**Categorical**	**Gamma model**	**Log-Normal model**	**Weibull model**	***PAF*_categorical - _*PAF*_gamma_**

Argentina	0.02488	0.08209	-0.06204	-0.04912	-0.03488	-0.04748	-0.01292
Australia	0.04	0.078	-0.06679	-0.05195	-0.03473	-0.05064	-0.01484
Austria	0.07014	0.15774	-0.03393	-0.0344	-0.03358	-0.03185	0.00047
Belize	0.20958	0.28647	0.01671	0.02142	0.02176	0.02153	-0.00471
Brazil	0.14516	0.2724	0.01291	0.01974	0.0221	0.01964	-0.00683
Canada	0.05019	0.1359	-0.04406	-0.03732	-0.02801	-0.03536	-0.00674
Costa Rica	0.07212	0.24279	-0.00984	0.00218	0.01119	0.00484	-0.01202
Czech Republic	0.02653	0.07235	-0.04347	-0.03926	-0.03536	-0.04003	-0.00421
Denmark	0.00669	0.02899	-0.08567	-0.08015	-0.06768	-0.0783	-0.00552
ECAS: Finland	0.06855	0	-0.08737	-0.0843	-0.07434	-0.08173	-0.00307
ECAS: France	0.12632	0	-0.07771	-0.06634	-0.05575	-0.06515	-0.01137
ECAS: Germany	0.11828	0	-0.08546	-0.07494	-0.06289	-0.073	-0.01052
ECAS: Italy	0.107	0	-0.08652	-0.07515	-0.06007	-0.07519	-0.01137
ECAS: Sweden	0.07803	0	-0.08431	-0.07873	-0.06794	-0.07597	-0.00558
ECAS: UK	0.10422	0	-0.06259	-0.04982	-0.05368	-0.04976	-0.01277
Finland	0.03181	0.05196	-0.07492	-0.06375	-0.04797	-0.06205	-0.01117
France	0.01975	0.2008	-0.02168	-0.0214	-0.01805	-0.02032	-0.00028
Germany	0.01415	0.03101	-0.08136	-0.07386	-0.05941	-0.07326	-0.0075
Hungary	0.04696	0.04052	-0.07032	-0.0531	-0.03804	-0.04984	-0.01722
Iceland	0.04117	0.09005	-0.06259	-0.05392	-0.04428	-0.05279	-0.00867
India	0.56138	0.10816	0.00549	0.00725	0.00506	0.0058	-0.00176
Ireland	0.16501	0.06958	-0.03008	-0.02443	-0.03142	-0.02394	-0.00565
Isle of Man	0.00885	0.06195	-0.05799	-0.0442	-0.03528	-0.04466	-0.01379
Israel	0.23209	0	-0.06315	-0.048	-0.03712	-0.04455	-0.01515
Italy	0.05808	0.03977	-0.07456	-0.06762	-0.05524	-0.06853	-0.00694
Japan	0.04869	0.04148	-0.06659	-0.04611	-0.03092	-0.04448	-0.02048
Kazakhstan	0.04267	0.21336	-0.01315	-0.0054	-0.0001	-0.00513	-0.00775
Mexico	0.09404	0.13644	-0.03411	-0.01966	-0.01217	-0.01758	-0.01445
Netherlands	0.06032	0.10269	-0.05643	-0.0539	-0.04592	-0.05264	-0.00253
Nicaragua	0.12052	0.45114	0.05098	0.0533	0.05278	0.05332	-0.00232
Nigeria	0.41863	0.18445	0.0144	0.0159	0.01485	0.01489	-0.0015
Norway	0.02321	0.06286	-0.06847	-0.06023	-0.04747	-0.05749	-0.00824
Peru	0.03488	0.14147	-0.04085	-0.02909	-0.02334	-0.02555	-0.01176
Spain	0.09172	0.23378	-0.0116	-0.0071	0.00214	-0.00672	-0.0045
Sri Lanka	0.19403	0.27032	0.01625	0.02531	0.02578	0.02476	-0.00906
Sweden	0.05049	0.06481	-0.06583	-0.04793	-0.04038	-0.0462	-0.0179
Switzerland	0.06763	0.0412	-0.07542	-0.07214	-0.06481	-0.06895	-0.00328
Uganda	0.28611	0.18889	0.01732	0.01764	0.01246	0.01555	-0.00032
Uruguay	0.04787	0.14096	-0.03916	-0.02318	-0.01523	-0.02209	-0.01598
USA 2	0.16125	0.1617	-0.02577	-0.01383	-0.0064	-0.01152	-0.01194
USA 3	0.26011	0.0707	-0.04069	-0.02818	-0.02105	-0.0261	-0.01251
USA 2	0.3263	0.18852	-0.11105	-0.06216	-0.05102	-0.05505	-0.04889
USA 3	0.38019	0.05805	-0.16022	-0.09638	-0.08313	-0.08908	-0.06384

**Table 8:** Proportion estimates for lifetime abstainers and former drinkers, as well as Population-Attributable Fraction (PAF) estimates for pancreatitis using a categorical model and continuous models (Gamma, Log-Normal, and Weibull) for women

	Proportions		PAF estimates				
**Country**	**Abstainers**	**Former drinkers**	**Categorical**	**Gamma model**	**Log-Normal model**	**Weibull model**	***PAF*_categorical - _*PAF*_gamma_**

Argentina	0.02488	0.08209	-0.06204	-0.04912	-0.03488	-0.04748	-0.01292
Australia	0.04	0.078	-0.06679	-0.05195	-0.03473	-0.05064	-0.01484
Austria	0.07014	0.15774	-0.03393	-0.0344	-0.03358	-0.03185	0.00047
Belize	0.20958	0.28647	0.01671	0.02142	0.02176	0.02153	-0.00471
Brazil	0.14516	0.2724	0.01291	0.01974	0.0221	0.01964	-0.00683
Canada	0.05019	0.1359	-0.04406	-0.03732	-0.02801	-0.03536	-0.00674
Costa Rica	0.07212	0.24279	-0.00984	0.00218	0.01119	0.00484	-0.01202
Czech Republic	0.02653	0.07235	-0.04347	-0.03926	-0.03536	-0.04003	-0.00421
Denmark	0.00669	0.02899	-0.08567	-0.08015	-0.06768	-0.0783	-0.00552
ECAS: Finland	0.06855	0	-0.08737	-0.0843	-0.07434	-0.08173	-0.00307
ECAS: France	0.12632	0	-0.07771	-0.06634	-0.05575	-0.06515	-0.01137
ECAS: Germany	0.11828	0	-0.08546	-0.07494	-0.06289	-0.073	-0.01052
ECAS: Italy	0.107	0	-0.08652	-0.07515	-0.06007	-0.07519	-0.01137
ECAS: Sweden	0.07803	0	-0.08431	-0.07873	-0.06794	-0.07597	-0.00558
ECAS: UK	0.10422	0	-0.06259	-0.04982	-0.05368	-0.04976	-0.01277
Finland	0.03181	0.05196	-0.07492	-0.06375	-0.04797	-0.06205	-0.01117
France	0.01975	0.2008	-0.02168	-0.0214	-0.01805	-0.02032	-0.00028
Germany	0.01415	0.03101	-0.08136	-0.07386	-0.05941	-0.07326	-0.0075
Hungary	0.04696	0.04052	-0.07032	-0.0531	-0.03804	-0.04984	-0.01722
Iceland	0.04117	0.09005	-0.06259	-0.05392	-0.04428	-0.05279	-0.00867
India	0.56138	0.10816	0.00549	0.00725	0.00506	0.0058	-0.00176
Ireland	0.16501	0.06958	-0.03008	-0.02443	-0.03142	-0.02394	-0.00565
Isle of Man	0.00885	0.06195	-0.05799	-0.0442	-0.03528	-0.04466	-0.01379
Israel	0.23209	0	-0.06315	-0.048	-0.03712	-0.04455	-0.01515
Italy	0.05808	0.03977	-0.07456	-0.06762	-0.05524	-0.06853	-0.00694
Japan	0.04869	0.04148	-0.06659	-0.04611	-0.03092	-0.04448	-0.02048
Kazakhstan	0.04267	0.21336	-0.01315	-0.0054	-0.0001	-0.00513	-0.00775
Mexico	0.09404	0.13644	-0.03411	-0.01966	-0.01217	-0.01758	-0.01445
Netherlands	0.06032	0.10269	-0.05643	-0.0539	-0.04592	-0.05264	-0.00253
Nicaragua	0.12052	0.45114	0.05098	0.0533	0.05278	0.05332	-0.00232
Nigeria	0.41863	0.18445	0.0144	0.0159	0.01485	0.01489	-0.0015
Norway	0.02321	0.06286	-0.06847	-0.06023	-0.04747	-0.05749	-0.00824
Peru	0.03488	0.14147	-0.04085	-0.02909	-0.02334	-0.02555	-0.01176
Spain	0.09172	0.23378	-0.0116	-0.0071	0.00214	-0.00672	-0.0045
Sri Lanka	0.19403	0.27032	0.01625	0.02531	0.02578	0.02476	-0.00906
Sweden	0.05049	0.06481	-0.06583	-0.04793	-0.04038	-0.0462	-0.0179
Switzerland	0.06763	0.0412	-0.07542	-0.07214	-0.06481	-0.06895	-0.00328
Uganda	0.28611	0.18889	0.01732	0.01764	0.01246	0.01555	-0.00032
Uruguay	0.04787	0.14096	-0.03916	-0.02318	-0.01523	-0.02209	-0.01598
USA 2	0.16125	0.1617	-0.02577	-0.01383	-0.0064	-0.01152	-0.01194
USA 3	0.26011	0.0707	-0.04069	-0.02818	-0.02105	-0.0261	-0.01251
USA 2	0.3263	0.18852	-0.11105	-0.06216	-0.05102	-0.05505	-0.04889
USA 3	0.38019	0.05805	-0.16022	-0.09638	-0.08313	-0.08908	-0.06384

**Table 9:** Proportion estimates for lifetime abstainers and former drinkers, as well as Population-Attributable Fraction (PAF) estimates for pancreatitis using a categorical model and continuous models (Gamma, Log-Normal, and Weibull) for men

	Proportions		PAF estimates				
**Country**	**Abstainers**	**Former drinkers**	**Categorical**	**Gamma model**	**Log-Normal model**	**Weibull model**	***PAF*_categorical - _*PAF*_gamma_**

Argentina	0.02488	0.08209	0.22296	0.15927	0.43723	0.20014	0.06369

Australia	0.04	0.078	0.08654	0.08482	0.38073	0.10451	0.00172

Austria	0.07014	0.15774	0.28936	0.22295	0.35679	0.2301	0.06641

Belize	0.20958	0.28647	0.33325	0.24985	0.33703	0.27395	0.0834

Brazil	0.14516	0.2724	0.36261	0.29194	0.39178	0.32264	0.07067

Canada	0.05019	0.1359	0.21733	0.13226	0.33792	0.15511	0.08507

Costa Rica	0.07212	0.24279	0.12691	0.10615	0.29474	0.14666	0.02076

Czech Republic	0.02653	0.07235	0.45021	0.44383	0.59431	0.46265	0.00638

Denmark	0.00669	0.02899	0.21317	0.1266	0.36293	0.13557	0.08657

ECAS: Finland	0.06855	0	0.15448	0.09867	0.28828	0.1085	0.05581

ECAS: France	0.12632	0	0.27896	0.19496	0.44157	0.22458	0.084

ECAS: Germany	0.11828	0	0.11683	0.07036	0.27456	0.07978	0.04647

ECAS: Italy	0.107	0	0.14476	0.13578	0.42278	0.1422	0.00898

ECAS: Sweden	0.07803	0	0.14909	0.04825	0.19429	0.05417	0.10084

ECAS: UK	0.10422	0	0.52217	0.49318	0.58899	0.50824	0.02899

Finland	0.03181	0.05196	0.12555	0.07766	0.33705	0.0898	0.04789

France	0.01975	0.2008	0.24059	0.22953	0.39322	0.24816	0.01106

Germany	0.01415	0.03101	0.18565	0.16192	0.44023	0.17239	0.02373

Hungary	0.04696	0.04052	0.08571	0.0504	0.29853	0.07345	0.03531

Iceland	0.04117	0.09005	0.0527	0.04367	0.15151	0.04482	0.00903

India	0.56138	0.10816	0.40834	0.36381	0.36933	0.36204	0.04453

Ireland	0.16501	0.06958	0.58943	0.51065	0.5712	0.52348	0.07878

Isle of Man	0.00885	0.06195	0.41981	0.3877	0.57684	0.42486	0.03211

Israel	0.23209	0	0.15418	0.05803	0.26452	0.09695	0.09615

Italy	0.05808	0.03977	0.13931	0.18626	0.45169	0.18221	-0.04695

Japan	0.04869	0.04148	0.25401	0.2705	0.53622	0.35619	-0.01649

Kazakhstan	0.04267	0.21336	0.42465	0.27561	0.43974	0.30884	0.14904

Mexico	0.09404	0.13644	0.29837	0.18325	0.36482	0.23945	0.11512

Netherlands	0.06032	0.10269	0.13115	0.11909	0.29457	0.12463	0.01206

Nicaragua	0.12052	0.45114	0.35959	0.24862	0.30616	0.26612	0.11097

Nigeria	0.41863	0.18445	0.35308	0.37914	0.43234	0.39043	-0.02606

Norway	0.02321	0.06286	0.13943	0.06723	0.2652	0.07835	0.0722

Peru	0.03488	0.14147	0.17245	0.04226	0.05929	0.04328	0.13019

Spain	0.09172	0.23378	0.22602	0.19353	0.43043	0.20917	0.03249

Sri Lanka	0.19403	0.27032	0.36577	0.31928	0.36454	0.33162	0.04649

Sweden	0.05049	0.06481	0.03966	0.02675	0.08574	0.02787	0.01291

Switzerland	0.06763	0.0412	0.25281	0.12628	0.30113	0.14007	0.12653

Uganda	0.28611	0.18889	0.56306	0.5551	0.55585	0.55323	0.00796

Uruguay	0.04787	0.14096	0.39759	0.24	0.43627	0.2895	0.15759

USA 2	0.16125	0.1617	0.18083	0.11673	0.28693	0.15662	0.0641

USA 3	0.26011	0.0707	0.2473	0.11757	0.28915	0.15812	0.12973

The alcohol PAF estimates that incorporated the Gamma and Weibull distributions are very similar and, for the most part, are within 1% of one another. Since the Log-Normal distribution is known to have a heavy tail, and this study includes data values for alcohol consumption up to 300 g/day, the alcohol PAF estimates from the Log-Normal distribution tend to be much larger and unrealistic when compared to the estimates from the Gamma and Weibull distributions.

Overall, the PAF estimates from the categorical model, Gamma model, and Weibull model are relatively similar when the survey data are more compact, but for those countries where data are more spread out, PAF estimates are more susceptible to sampling bias for diseases with a relatively linear or exponential risk relationship with alcohol, such as pancreatitis and breast cancer. For example, for Brazilian men the alcohol consumption prevalence data tend to be very spread out when compared to men from France, leading to a small difference in the PAFs for pancreatitis. However, this trend does not apply when we look at a disease, such as diabetes, that has a J-shaped relative risk function. If we look at the same example, we find that the alcohol PAFs for diabetes provide similar estimates from the categorical model, Gamma model, Log-Normal model, and Weibull model for men from both Brazil and France. This is due to the fact that the relative risk functions are exponential for pancreatitis and are J-shaped for diabetes and thus have different properties. The J-shaped curve in some cases leads to a negative PAF (which represents the fraction of deaths prevented) as the risk of diabetes at the population level is less under current levels of alcohol consumption than under the counterfactual scenario of no alcohol consumption.

### Characterizing the alcohol consumption gamma distribution

Based on data from GENACIS and STEPS, the mean daily average per capita alcohol consumption among drinkers was estimated to be 7.549 grams for women (the Gamma standard deviation was 9.862) and 18.292 grams for men (the Gamma standard deviation was 22.015) (see Table 10).

**Table 10:** Descriptive statistics of the alcohol surveys from 66 countries

	Number of estimates	Empirical mean	Empirical standard deviation	Gamma distribution mean	Gamma distribution standard deviation
Women	422	7.55	12.63	7.55	9.86

Men	429	18.29	25.60	18.29	22.01

Total	851	12.96	19.17	12.96	15.99

After analyzing the association between the Gamma mean and the Gamma standard deviation, a strong linear relationship was established. Analysis of the residuals of various general linear models led to the conclusion that a general linear model with a normal distribution and an identity link (i.e., a linear regression model) is the best possible model to characterize the relationship between the standard deviation of the Gamma distribution and the mean of the Gamma distribution. As a statistical interaction was determined to be present by gender for the relationship between the Gamma mean and the Gamma standard deviation, this linear relationship was modeled separately for men and for women.

**Figure 7:**
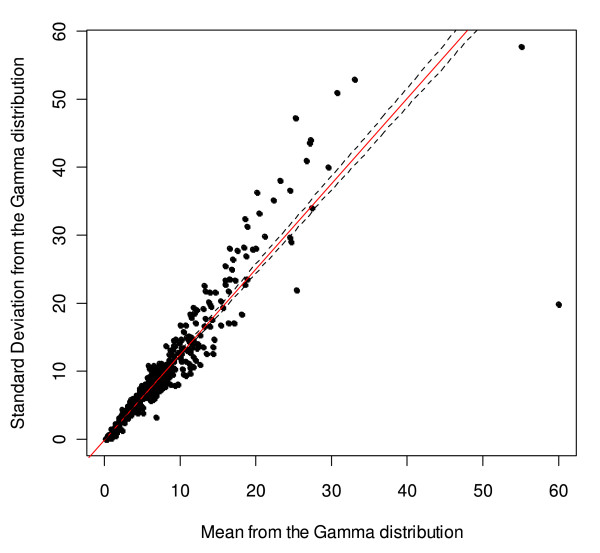
**Regression analysis and scatter plot for the mean and standard deviation of the alcohol consumption Gamma distribution for women**.

**Figure 8:**
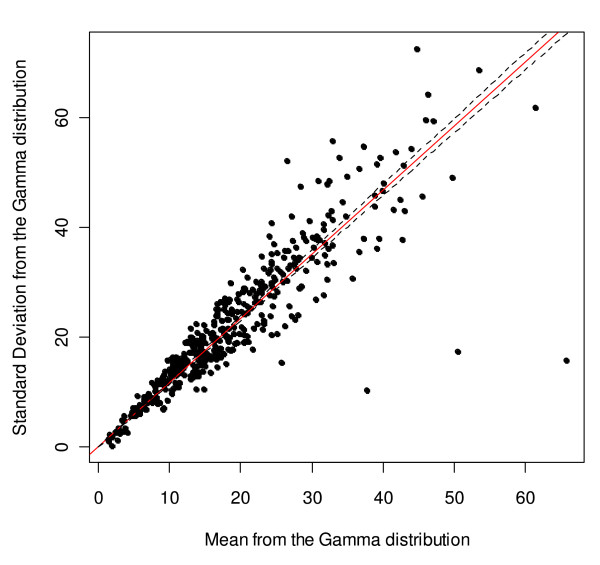
**Regression analysis and scatter plot for the mean and standard deviation of the alcohol consumption Gamma distribution for men**.

Figures 7 and 8 illustrate the linear fit for women and men, respectively. The linear regressions indicate that a unit increase in mean alcohol consumption is associated with an increase of 1.258 (95% CI: 1.223 to 1.293) in the standard deviation of the Gamma alcohol consumption distribution for women and 1.171 (95% CI: 1.144 to 1.197) in the standard deviation of the Gamma alcohol consumption distribution for men. Additionally, for women the linear regression indicated that 92.07% of the variation of the standard deviation of the Gamma distribution was explained by the mean, while for men 94.74% of the variation of the standard deviation of the Gamma distribution was explained by the mean.

Regression diagnostics indicated that there were some outliers. For women, two data points from Nigeria and one from Uganda were identified as influential observations, while for men, two observations in Germany and one in Nigeria were identified as influential observations. There was no indication of a lack of homoscedasticity for any of the regression models (Additional file 1).

## Discussion

Both the Gamma and the Weibull distributions summarized the population distribution of average volume of alcohol consumption more accurately than did the Log-Normal distribution. Moreover, for the Gamma and Weibull distributions the ratio of mean to standard deviation was comparable across all countries, irrespective of drinking patterns and the survey measure used to measure alcohol consumption. Overall, both the Gamma and Weibull distributions yield similar PAFs and could be used in descriptive alcohol epidemiology. Although not examined specifically, these outcomes would also apply to PAFs that are calculated when using a counterfactual scenario where alcohol consumption is decreased due to a policy or intervention such as taxation. Since the Weibull distribution is a more complicated distribution and less flexible than the Gamma distribution, and since it is possible to shift the Gamma distribution upwards (necessary in modeling the burden of disease attributable to alcohol consumption), the Gamma distribution is the best distribution for modeling alcohol consumption.

Modeling survey alcohol consumption data alone without correcting the distribution for undercoverage will lead to inaccurate alcohol PAFs as self-reported survey data typically underestimate alcohol consumption based on sales or taxation (e.g., [26]). In other words, alcohol surveys often do not accurately represent the population due to undercoverage where some members of the population are inadequately represented (or excluded) or due to response bias [30]. Accordingly, a method must be developed that will shift the exposure distribution so that it is consistent with per capita consumption data in order to correct for survey bias and allow for a more accurate estimation of the true alcohol consumption distribution and for an accurate comparison of the alcohol-attributable burden of disease across countries.

Given the relationship between the mean and the standard deviation of alcohol consumption [15], modeling alcohol consumption using the Gamma distribution, up-estimating this distribution using the relationship between the mean and the standard deviation, and using per capita consumption data, allows us to correct for the biases that lead to undercoverage (for specifics on the upshifting methods see [15]) and allows for the estimation of the distribution of alcohol consumption in a country as if it were measured by a survey with a much higher coverage rate. Additionally, based on the relationship between the mean and the standard deviation of the alcohol consumption Gamma distribution, we can use the mean alcohol consumption from sales and taxation data to obtain the κ and θ parameters for the alcohol exposure distribution for those countries where no survey data exist. Due to great variations in the populations surveyed, and in the sampling frame, response rate, and coverage rate for each of the individual surveys within the main survey groups of GENACIS, ECAS, and STEPS, our observations that alcohol consumption can best be modeled through a Gamma distribution and that the mean is highly correlated with the standard deviation of the alcohol consumption Gamma distribution indicate that these results are applicable to a wide range of countries and are valid for population surveys that use different methodologies.

An interesting finding from our study was the identification as outliers of some of the observations from Nigeria. This could be due to multiple factors. The number of observations from Nigeria upon which the mean and the standard deviation of the alcohol consumption Gamma distribution are based are fewer than the number of observations from other countries. A further factor is that the relationship between the mean and standard deviation of the alcohol consumption Gamma distribution for Nigeria may be different when compared to other countries. Given that only some age groups in Nigeria were identified by the regression diagnostics as outliers, it is very likely that these outliers were due to the low number of individuals surveyed in Nigeria. Future research will focus on modeling alcohol consumption by global region (such as by using the 2005 Comparative Risk Assessment regions [44]) to see if there are regional differences in the relationship between the mean and the standard deviation of the alcohol consumption Gamma distribution.

## Conclusion

When comparing the Log-Normal, Weibull, and Gamma distributions to calculate average consumption of alcohol, the Gamma distribution and the Weibull distribution outperform the Log-Normal distribution in fitting the empirical consumption distribution. Of these two distributions, the Gamma distribution appears to be the best choice for modeling as it has two parameters that can easily be shifted to make the fit more compatible with the per capita consumption data, thus making it possible to estimate the exposure distribution of countries with only aggregate per capita consumption reported, as long as prevalence of abstention is known (see [15]). Thus, shifting the mean upwards is possible, as the Gamma distribution can be described by two parameters (mean and standard deviation), which empirically can be reduced to one, as a large degree of variance of the standard deviation of the alcohol consumption Gamma distribution is explained by the mean alcohol consumption. Accurate modeling of alcohol consumption as an upshifted distribution will provide public health decision-makers with accurate data to assess the impact of alcohol consumption within and across countries and will aid in determining public health priorities and where to allocate resources.

## Competing interests

The authors declare that they have no competing interests.

## Authors' contributions

TK, GG, and JR conceptualized the overall article. TK, GG, KDS, GG, and JR contributed to the methodology, identified sources for risk relations and exposure, and contributed to the writing. TK performed all statistical analyses. All authors have approved the final version.

## Supplementary Material

Additional file 1 **Web Appendix**. This web appendix includes parameter estimates using non-truncated data for women and men from Log-Normal, Gamma, and Weibull models, proportion estimates for lifetime abstainers and former drinkers, as well as Population Attributable Fraction (PAF) estimates for breast cancer, diabetes, pancreatitis using a categorical model and a continuous model. Count, proportion and weighted global proportion estimates for women and men drinkers that drink ≤ 96 g/day, > 96 g/day, ≤ 120 g/day, and > 120 g/day were also included. Proportion estimates for the decomposition of alcohol Population Attributable Fraction (PAF) are listed for breast cancer and pancreatitis consisting of drinkers that drink ≤ 96 g/day and > 96 g/day, ≤ 120 g/day and > 120 g/day, ≤ 150 g/day and > 150 g/day, and ≤ 200 g/day and > 200 g/day using a continuous model (Gamma, Log-Normal, and Weibull) for women and men. Click here for file
