# Supplementary material for: Determining the best population-level alcohol consumption model and its impact on estimates of alcohol-attributable harms
Source: Popul Health Metr. 2012 Apr 10;10:6. doi: 10.1186/1478-7954-10-6 (PMC3352241; doi:10.1186/1478-7954-10-6)
Supplement: Additional file 1 — Web Appendix. This web appendix includes parameter estimates using non-truncated data for women and men from Log-Normal, Gamma, and Weibull models, proportion estimates for lifetime abstainers and former drinkers, as well as Population Attributable Fraction (PAF) estimates for breast cancer, diabetes, pancreatitis using a categorical model and a continuous model. Count, proportion and weighted global proportion estimates for women and men drinkers that drink ≤ 96 g/day, > 96 g/day, ≤ 120 g/day, and > 120 g/day were also included. Proportion estimates for the decomposition of alcohol Population Attributable Fraction (PAF) are listed for breast cancer and pancreatitis consisting of drinkers that drink ≤ 96 g/day and > 96 g/day, ≤ 120 g/day and > 120 g/day, ≤ 150 g/day and > 150 g/day, and ≤ 200 g/day and > 200 g/day using a continuous model (Gamma, Log-Normal, and Weibull) for women and men. [file 1478-7954-10-6-S1.DOC]

Table 1. Parameter Estimates from Log-Normal, Gamma, and Weibull models for women not truncated from 41 data sets

|  | Log-Normal Model | | Gamma Model | | Weibull Model | |
| --- | --- | --- | --- | --- | --- | --- |
|  | Parameter Estimates | | Parameter Estimates | | Parameter Estimates | |
| Country | Mean | Standard Deviation | Scale | Shape | Scale | Shape |
| Argentina | 0.14 | 1.93 | 9.17 | 0.48 | 2.92 | 0.60 |
| Australia | 0.57 | 1.88 | 11.75 | 0.51 | 4.33 | 0.64 |
| Australia 1 | 0.47 | 1.57 | 8.57 | 0.56 | 3.55 | 0.67 |
| Austria | 1.91 | 0.92 | 8.45 | 1.26 | 10.85 | 1.05 |
| Belize | 0.64 | 1.51 | 13.44 | 0.50 | 4.17 | 0.62 |
| Brazil | 1.09 | 2.10 | 36.30 | 0.41 | 8.18 | 0.54 |
| Canada | 1.06 | 1.41 | 9.92 | 0.69 | 5.78 | 0.77 |
| Costa Rica | -0.28 | 1.81 | 7.20 | 0.45 | 1.88 | 0.57 |
| Czech Republic | 1.04 | 1.70 | 16.47 | 0.54 | 6.49 | 0.66 |
| Denmark | 1.37 | 1.40 | 9.37 | 0.84 | 7.46 | 0.89 |
| ECAS: Finland | 1.07 | 1.20 | 6.51 | 0.88 | 5.26 | 0.87 |
| ECAS: France | 0.94 | 1.56 | 10.94 | 0.63 | 5.51 | 0.72 |
| ECAS: Germany | 1.05 | 1.35 | 10.11 | 0.69 | 5.54 | 0.76 |
| ECAS: Italy | 1.37 | 1.59 | 15.91 | 0.64 | 8.45 | 0.74 |
| ECAS: Sweden | 0.90 | 1.17 | 4.23 | 1.02 | 4.30 | 0.99 |
| ECAS: UK | 1.70 | 1.48 | 19.03 | 0.69 | 11.13 | 0.77 |
| Finland | 0.47 | 1.67 | 7.08 | 0.61 | 3.47 | 0.72 |
| France | 1.62 | 1.05 | 9.30 | 0.98 | 8.75 | 0.92 |
| Germany | 1.30 | 1.47 | 12.42 | 0.70 | 7.43 | 0.78 |
| Hungary | -0.82 | 1.89 | 4.36 | 0.44 | 1.11 | 0.58 |
| Iceland | 0.82 | 1.31 | 5.78 | 0.81 | 4.23 | 0.84 |
| India | 1.31 | 2.16 | 42.29 | 0.42 | 10.39 | 0.55 |
| Ireland | 2.01 | 1.23 | 15.55 | 0.91 | 13.53 | 0.91 |
| Isle of Man | 1.18 | 1.85 | 16.98 | 0.57 | 7.59 | 0.69 |
| Israel | -0.05 | 1.98 | 12.55 | 0.40 | 2.52 | 0.54 |
| Italy | 1.52 | 1.39 | 12.97 | 0.77 | 8.95 | 0.83 |
| Japan | -0.15 | 2.18 | 14.32 | 0.37 | 2.53 | 0.50 |
| Kazakhstan | -0.52 | 1.93 | 6.67 | 0.42 | 1.52 | 0.56 |
| Mexico | -1.15 | 1.63 | 5.03 | 0.37 | 0.76 | 0.53 |
| Netherlands | 1.44 | 1.11 | 8.33 | 0.94 | 7.43 | 0.91 |
| Nicaragua | 0.91 | 1.49 | 26.83 | 0.43 | 5.54 | 0.57 |
| Nigeria | 1.84 | 2.31 | 65.85 | 0.43 | 18.29 | 0.56 |
| Norway | 0.61 | 1.58 | 7.07 | 0.66 | 3.85 | 0.75 |
| Peru | 0.16 | 0.91 | 1.62 | 1.18 | 1.89 | 0.98 |
| Spain | 1.07 | 1.78 | 13.31 | 0.61 | 6.58 | 0.72 |
| Sri Lanka | -2.28 | 1.69 | 3.31 | 0.30 | 0.27 | 0.46 |
| Sweden | 0.44 | 1.26 | 4.15 | 0.79 | 2.93 | 0.83 |
| Switzerland | 1.39 | 1.25 | 8.07 | 0.93 | 7.21 | 0.93 |
| Uganda | 0.98 | 2.09 | 34.50 | 0.40 | 7.39 | 0.53 |
| Uruguay | 0.19 | 1.90 | 11.60 | 0.45 | 3.10 | 0.58 |
| USA 1 | 0.18 | 1.96 | 12.42 | 0.43 | 3.16 | 0.56 |
| USA 2 | 0.30 | 1.62 | 11.49 | 0.47 | 3.12 | 0.59 |
| USA 3 | 0.23 | 1.67 | 9.85 | 0.48 | 2.94 | 0.61 |

Table 2. Parameter Estimates from Log-Normal, Gamma, and Weibull models for men not truncated at 300 g / day from 41 data sets

|  | Log-Normal Model | | Gamma Model | | Weibull Model | |
| --- | --- | --- | --- | --- | --- | --- |
|  | Parameter Estimates | | Parameter Estimates | | Parameter Estimates | |
| Country | Mean | Standard Deviation | Scale | Shape | Scale | Shape |
| Argentina | 1.84 | 1.68 | 25.33 | 0.64 | 13.62 | 0.75 |
| Australia | 1.63 | 1.69 | 18.79 | 0.67 | 10.99 | 0.78 |
| Austria | 2.85 | 0.96 | 19.72 | 1.33 | 27.52 | 1.13 |
| Belize | 2.06 | 1.55 | 38.32 | 0.59 | 16.87 | 0.69 |
| Brazil | 1.57 | 2.01 | 47.07 | 0.44 | 12.55 | 0.57 |
| Canada | 1.96 | 1.42 | 21.83 | 0.74 | 14.05 | 0.80 |
| Costa Rica | 1.13 | 1.87 | 23.50 | 0.49 | 7.71 | 0.61 |
| Czech Republic | 2.58 | 1.55 | 38.84 | 0.75 | 26.59 | 0.83 |
| Denmark | 2.28 | 1.24 | 18.05 | 0.98 | 17.33 | 0.96 |
| ECAS: Finland | 2.22 | 1.18 | 16.68 | 0.99 | 16.13 | 0.95 |
| ECAS: France | 2.18 | 1.48 | 26.46 | 0.75 | 17.58 | 0.82 |
| ECAS: Germany | 1.92 | 1.33 | 16.43 | 0.84 | 12.84 | 0.87 |
| ECAS: Italy | 2.22 | 1.40 | 20.68 | 0.87 | 17.43 | 0.92 |
| ECAS: Sweden | 1.79 | 1.26 | 13.48 | 0.87 | 10.94 | 0.88 |
| ECAS: UK | 2.85 | 1.31 | 41.58 | 0.84 | 32.09 | 0.86 |
| Finland | 1.76 | 1.51 | 17.08 | 0.75 | 11.58 | 0.83 |
| France | 2.44 | 1.25 | 25.29 | 0.88 | 21.08 | 0.90 |
| Germany | 2.27 | 1.37 | 21.29 | 0.88 | 18.07 | 0.92 |
| Hungary | 1.10 | 1.81 | 17.13 | 0.55 | 6.95 | 0.67 |
| Iceland | 1.64 | 1.25 | 9.84 | 0.96 | 9.17 | 0.95 |
| India | 2.25 | 1.95 | 71.22 | 0.49 | 23.85 | 0.61 |
| Ireland | 3.04 | 1.18 | 38.57 | 0.98 | 36.94 | 0.95 |
| Isle of Man | 2.22 | 1.79 | 40.15 | 0.62 | 20.55 | 0.73 |
| Israel | 1.02 | 1.87 | 22.11 | 0.48 | 6.85 | 0.61 |
| Italy | 2.44 | 1.30 | 21.80 | 0.96 | 20.92 | 0.99 |
| Japan | 1.63 | 2.19 | 37.45 | 0.49 | 13.60 | 0.63 |
| Kazakhstan | 1.87 | 1.76 | 37.66 | 0.55 | 14.73 | 0.66 |
| Mexico | 1.34 | 1.90 | 33.59 | 0.46 | 9.69 | 0.59 |
| Netherlands | 2.28 | 1.17 | 17.45 | 1.00 | 17.27 | 0.98 |
| Nicaragua | 2.03 | 1.52 | 38.43 | 0.58 | 16.28 | 0.68 |
| Nigeria | 2.47 | 1.79 | 56.65 | 0.59 | 27.03 | 0.71 |
| Norway | 1.66 | 1.44 | 15.92 | 0.74 | 10.25 | 0.80 |
| Peru | 1.13 | 1.17 | 8.89 | 0.76 | 5.60 | 0.79 |
| Spain | 2.28 | 1.49 | 25.30 | 0.81 | 19.04 | 0.87 |
| Sri Lanka | 1.30 | 2.18 | 58.77 | 0.37 | 10.73 | 0.51 |
| Sweden | 1.12 | 1.32 | 8.20 | 0.79 | 5.83 | 0.83 |
| Switzerland | 2.37 | 1.12 | 17.67 | 1.05 | 18.27 | 0.97 |
| Uganda | 2.75 | 1.79 | 70.44 | 0.61 | 35.46 | 0.72 |
| Uruguay | 1.69 | 1.84 | 35.30 | 0.52 | 12.89 | 0.64 |
| USA 2 | 1.41 | 1.72 | 25.65 | 0.53 | 9.50 | 0.64 |
| USA 3 | 1.33 | 1.81 | 29.43 | 0.48 | 9.06 | 0.61 |

Table 3. Proportion estimates for lifetime abstainers and former drinkers, as well as Population Attributable Fraction (PAF) estimates for breast cancer using a categorical model and a continuous model (Gamma, Log-Normal, and Weibull) for women not truncated at 300 g / day

|  | Proportions | | PAF Estimates | | | |
| --- | --- | --- | --- | --- | --- | --- |
| Country | Abstainers | Former Drinkers | Categorical | Gamma Model | Log-Normal Model | Weibull Model |
| Argentina | 0.06355 | 0.29933 | 0.14923 | 0.13540 | 0.15384 | 0.13620 |
| Australia | 0.04951 | 0.13319 | 0.10734 | 0.09444 | 0.12996 | 0.09600 |
| Australia 1 | 0.12341 | 0.06414 | 0.08152 | 0.06024 | 0.07021 | 0.05996 |
| Austria | 0.17941 | 0.32880 | 0.16652 | 0.16302 | 0.16320 | 0.16338 |
| Belize | 0.59903 | 0.21449 | 0.10145 | 0.09595 | 0.09592 | 0.09510 |
| Brazil | 0.22741 | 0.36006 | 0.19526 | 0.18374 | 0.20113 | 0.18618 |
| Canada | 0.09532 | 0.16116 | 0.11890 | 0.10649 | 0.11779 | 0.10619 |
| Costa Rica | 0.19253 | 0.37923 | 0.16137 | 0.15162 | 0.15593 | 0.15135 |
| Czech Republic | 0.05538 | 0.14665 | 0.13325 | 0.11822 | 0.14643 | 0.11888 |
| Denmark | 0.00971 | 0.07061 | 0.10045 | 0.09091 | 0.12153 | 0.09110 |
| ECAS: Finland | 0.07480 | 0.00197 | 0.06369 | 0.04740 | 0.05292 | 0.04699 |
| ECAS: France | 0.27238 | 0.00000 | 0.05939 | 0.04492 | 0.06499 | 0.04519 |
| ECAS: Germany | 0.18471 | 0.00000 | 0.07463 | 0.05066 | 0.05713 | 0.04848 |
| ECAS: Italy | 0.21206 | 0.00195 | 0.08903 | 0.07422 | 0.11217 | 0.07535 |
| ECAS: Sweden | 0.13200 | 0.00200 | 0.04803 | 0.03386 | 0.03994 | 0.03388 |
| ECAS: UK | 0.14286 | 0.00000 | 0.11594 | 0.10312 | 0.13957 | 0.10394 |
| Finland | 0.06491 | 0.04057 | 0.06973 | 0.05056 | 0.07463 | 0.05056 |
| France | 0.03552 | 0.41525 | 0.19287 | 0.18747 | 0.18762 | 0.18745 |
| Germany | 0.02588 | 0.03691 | 0.10094 | 0.08681 | 0.11568 | 0.08677 |
| Hungary | 0.17718 | 0.05964 | 0.06264 | 0.03701 | 0.04530 | 0.03630 |
| Iceland | 0.07396 | 0.08261 | 0.08375 | 0.06784 | 0.07546 | 0.06750 |
| India | 0.84014 | 0.10204 | 0.05502 | 0.05365 | 0.05764 | 0.05469 |
| Ireland | 0.23933 | 0.05937 | 0.11810 | 0.11259 | 0.13450 | 0.11288 |
| Isle of Man | 0.01838 | 0.11949 | 0.12723 | 0.11850 | 0.17570 | 0.12157 |
| Israel | 0.42882 | 0.00000 | 0.04408 | 0.02458 | 0.03910 | 0.02325 |
| Italy | 0.19622 | 0.06094 | 0.10517 | 0.08990 | 0.11227 | 0.09029 |
| Japan | 0.15058 | 0.08415 | 0.08860 | 0.06775 | 0.09511 | 0.06877 |
| Kazakhstan | 0.10143 | 0.26307 | 0.13401 | 0.11568 | 0.12401 | 0.11479 |
| Mexico | 0.40553 | 0.17212 | 0.09092 | 0.07588 | 0.07463 | 0.07449 |
| Netherlands | 0.14467 | 0.17495 | 0.12055 | 0.11305 | 0.11531 | 0.11294 |
| Nicaragua | 0.50282 | 0.39336 | 0.16442 | 0.15622 | 0.15366 | 0.15459 |
| Nigeria | 0.56034 | 0.22980 | 0.16004 | 0.15402 | 0.16134 | 0.15696 |
| Norway | 0.04049 | 0.07570 | 0.08266 | 0.06620 | 0.08621 | 0.06621 |
| Peru | 0.08966 | 0.29951 | 0.13924 | 0.12450 | 0.12399 | 0.12449 |
| Spain | 0.22908 | 0.32427 | 0.15470 | 0.15056 | 0.17495 | 0.15131 |
| Sri Lanka | 0.86610 | 0.06949 | 0.03264 | 0.03012 | 0.02992 | 0.02995 |
| Sweden | 0.09666 | 0.11230 | 0.08539 | 0.06797 | 0.06996 | 0.06777 |
| Switzerland | 0.19806 | 0.06082 | 0.08298 | 0.07341 | 0.08702 | 0.07340 |
| Uganda | 0.36412 | 0.26649 | 0.15735 | 0.14658 | 0.16190 | 0.14889 |
| Uruguay | 0.17308 | 0.22596 | 0.12907 | 0.11302 | 0.12813 | 0.11289 |
| USA 1 | 0.10302 | 0.13854 | 0.10544 | 0.08900 | 0.11203 | 0.08978 |
| USA 2 | 0.32630 | 0.18852 | 0.11255 | 0.09623 | 0.09806 | 0.09469 |
| USA 3 | 0.38019 | 0.05805 | 0.06399 | 0.04719 | 0.05318 | 0.04608 |

Table 4. Proportion estimates for lifetime abstainers and former drinkers, as well as Population Attributable Fractions (PAF) estimates for diabetes using a categorical model and continuous model (Gamma, Log-Normal, and Weibull) for women not truncated at 300 g / day

|  | Proportions | | PAF Estimates | | | |
| --- | --- | --- | --- | --- | --- | --- |
| Country | Abstainers | Former Drinkers | Categorical | Gamma Model | Log-Normal Model | Weibull Model |
| Argentina | 0.06355 | 0.29933 | -0.14692 | -0.06787 | -0.05274 | -0.06333 |
| Australia | 0.04951 | 0.13319 | -0.24721 | -0.15762 | -0.12626 | -0.15022 |
| Australia 1 | 0.12341 | 0.06414 | -0.26990 | -0.16237 | -0.14117 | -0.15421 |
| Austria | 0.17941 | 0.32880 | -0.10003 | -0.09967 | -0.09292 | -0.09467 |
| Belize | 0.59903 | 0.21449 | -0.01794 | -0.00596 | -0.00215 | -0.00340 |
| Brazil | 0.22741 | 0.36006 | -0.05408 | -0.02384 | -0.01732 | -0.02246 |
| Canada | 0.09532 | 0.16116 | -0.21615 | -0.16259 | -0.13959 | -0.15557 |
| Costa Rica | 0.19253 | 0.37923 | -0.06097 | -0.00801 | -0.00214 | -0.00435 |
| Czech Republic | 0.05538 | 0.14665 | -0.23423 | -0.17322 | -0.14215 | -0.16336 |
| Denmark | 0.00971 | 0.07061 | -0.33046 | -0.26493 | -0.22239 | -0.26241 |
| ECAS: Finland | 0.07480 | 0.00197 | -0.32158 | -0.25101 | -0.22964 | -0.24296 |
| ECAS: France | 0.27238 | 0.00000 | -0.25029 | -0.18164 | -0.15523 | -0.17478 |
| ECAS: Germany | 0.18471 | 0.00000 | -0.27970 | -0.21632 | -0.19179 | -0.20417 |
| ECAS: Italy | 0.21206 | 0.00195 | -0.28560 | -0.21937 | -0.18260 | -0.21217 |
| ECAS: Sweden | 0.13200 | 0.00200 | -0.29211 | -0.20986 | -0.19889 | -0.20854 |
| ECAS: UK | 0.14286 | 0.00000 | -0.30489 | -0.25529 | -0.22162 | -0.24719 |
| Finland | 0.06491 | 0.04057 | -0.29456 | -0.18499 | -0.16109 | -0.18021 |
| France | 0.03552 | 0.41525 | -0.10183 | -0.08985 | -0.08062 | -0.08479 |
| Germany | 0.02588 | 0.03691 | -0.33041 | -0.26864 | -0.22619 | -0.25848 |
| Hungary | 0.17718 | 0.05964 | -0.22547 | -0.08457 | -0.08059 | -0.07914 |
| Iceland | 0.07396 | 0.08261 | -0.26082 | -0.18598 | -0.16929 | -0.18056 |
| India | 0.84014 | 0.10204 | 0.00370 | 0.00412 | 0.00483 | 0.00417 |
| Ireland | 0.23933 | 0.05937 | -0.21416 | -0.21209 | -0.18943 | -0.20603 |
| Isle of Man | 0.01838 | 0.11949 | -0.27038 | -0.20513 | -0.15778 | -0.19787 |
| Israel | 0.42882 | 0.00000 | -0.17003 | -0.09966 | -0.08491 | -0.09059 |
| Italy | 0.19622 | 0.06094 | -0.25978 | -0.20770 | -0.17787 | -0.20246 |
| Japan | 0.15058 | 0.08415 | -0.23347 | -0.11957 | -0.09699 | -0.10624 |
| Kazakhstan | 0.10143 | 0.26307 | -0.14039 | -0.04881 | -0.04103 | -0.04260 |
| Mexico | 0.40553 | 0.17212 | -0.08857 | -0.02026 | -0.01479 | -0.01363 |
| Netherlands | 0.14467 | 0.17495 | -0.18932 | -0.16501 | -0.15025 | -0.15897 |
| Nicaragua | 0.50282 | 0.39336 | 0.02923 | 0.03438 | 0.03458 | 0.03517 |
| Nigeria | 0.56034 | 0.22980 | -0.00892 | 0.00013 | -0.00065 | -0.00187 |
| Norway | 0.04049 | 0.07570 | -0.28376 | -0.18535 | -0.16146 | -0.18067 |
| Peru | 0.08966 | 0.29951 | -0.12528 | -0.04802 | -0.04493 | -0.04558 |
| Spain | 0.22908 | 0.32427 | -0.07944 | -0.05418 | -0.03553 | -0.05184 |
| Sri Lanka | 0.86610 | 0.06949 | -0.00659 | 0.00516 | 0.00598 | 0.00625 |
| Sweden | 0.09666 | 0.11230 | -0.23299 | -0.13662 | -0.12713 | -0.13302 |
| Switzerland | 0.19806 | 0.06082 | -0.24288 | -0.20449 | -0.18102 | -0.20067 |
| Uganda | 0.36412 | 0.26649 | -0.05139 | -0.02926 | -0.02312 | -0.02722 |
| Uruguay | 0.17308 | 0.22596 | -0.14518 | -0.07583 | -0.06006 | -0.06876 |
| USA 1 | 0.10302 | 0.13854 | -0.22157 | -0.12244 | -0.10000 | -0.11227 |
| USA 2 | 0.32630 | 0.18852 | -0.11105 | -0.06216 | -0.05102 | -0.05505 |
| USA 3 | 0.38019 | 0.05805 | -0.16022 | -0.09638 | -0.08313 | -0.08908 |

Table 5. Proportion estimates for lifetime abstainers and former drinkers, as well as Population Attributable Fraction (PAF) estimates for diabetes using a categorical model and a continuous model (Gamma, Log-Normal, and Weibull) for men

|  | Proportions | | PAF Estimates | | | |
| --- | --- | --- | --- | --- | --- | --- |
| Country | Abstainers | Former Drinkers | Categorical | Gamma Model | Log-Normal Model | Weibull Model |
| Argentina | 0.02488 | 0.08209 | -0.06204 | -0.04912 | -0.03488 | -0.04748 |
| Australia | 0.04000 | 0.07800 | -0.06679 | -0.05195 | -0.03473 | -0.05064 |
| Austria | 0.07014 | 0.15774 | -0.03393 | -0.03440 | -0.03358 | -0.03185 |
| Belize | 0.20958 | 0.28647 | 0.01671 | 0.02169 | 0.02178 | 0.02172 |
| Brazil | 0.14516 | 0.27240 | 0.01291 | 0.01974 | 0.02210 | 0.01964 |
| Canada | 0.05019 | 0.13590 | -0.04406 | -0.03714 | -0.02799 | -0.03515 |
| Costa Rica | 0.07212 | 0.24279 | -0.00984 | 0.00218 | 0.01119 | 0.00484 |
| Czech Republic | 0.02653 | 0.07235 | -0.04347 | -0.03926 | -0.03536 | -0.04003 |
| Denmark | 0.00669 | 0.02899 | -0.08567 | -0.08015 | -0.06768 | -0.07830 |
| ECAS: Finland | 0.06855 | 0.00000 | -0.08737 | -0.08430 | -0.07434 | -0.08173 |
| ECAS: France | 0.12632 | 0.00000 | -0.07771 | -0.06603 | -0.05572 | -0.06481 |
| ECAS: Germany | 0.11828 | 0.00000 | -0.08546 | -0.07494 | -0.06289 | -0.07300 |
| ECAS: Italy | 0.10700 | 0.00000 | -0.08652 | -0.07515 | -0.06007 | -0.07519 |
| ECAS: Sweden | 0.07803 | 0.00000 | -0.08431 | -0.07873 | -0.06794 | -0.07597 |
| ECAS: UK | 0.10422 | 0.00000 | -0.06259 | -0.04622 | -0.05320 | -0.04666 |
| Finland | 0.03181 | 0.05196 | -0.07492 | -0.06375 | -0.04797 | -0.06205 |
| France | 0.01975 | 0.20080 | -0.02168 | -0.02140 | -0.01805 | -0.02032 |
| Germany | 0.01415 | 0.03101 | -0.08136 | -0.07386 | -0.05941 | -0.07326 |
| Hungary | 0.04696 | 0.04052 | -0.07032 | -0.05310 | -0.03804 | -0.04984 |
| Iceland | 0.04117 | 0.09005 | -0.06259 | -0.05392 | -0.04428 | -0.05279 |
| India | 0.56138 | 0.10816 | 0.00549 | 0.00758 | 0.00509 | 0.00598 |
| Ireland | 0.16501 | 0.06958 | -0.03008 | -0.02443 | -0.03142 | -0.02394 |
| Isle of Man | 0.00885 | 0.06195 | -0.05799 | -0.04348 | -0.03523 | -0.04407 |
| Israel | 0.23209 | 0.00000 | -0.06315 | -0.04800 | -0.03712 | -0.04455 |
| Italy | 0.05808 | 0.03977 | -0.07456 | -0.06762 | -0.05524 | -0.06853 |
| Japan | 0.04869 | 0.04148 | -0.06659 | -0.04611 | -0.03092 | -0.04448 |
| Kazakhstan | 0.04267 | 0.21336 | -0.01315 | -0.00486 | -0.00006 | -0.00477 |
| Mexico | 0.09404 | 0.13644 | -0.03411 | -0.01947 | -0.01215 | -0.01747 |
| Netherlands | 0.06032 | 0.10269 | -0.05643 | -0.05390 | -0.04592 | -0.05264 |
| Nicaragua | 0.12052 | 0.45114 | 0.05098 | 0.05330 | 0.05278 | 0.05332 |
| Nigeria | 0.41863 | 0.18445 | 0.01440 | 0.01610 | 0.01487 | 0.01503 |
| Norway | 0.02321 | 0.06286 | -0.06847 | -0.06023 | -0.04747 | -0.05749 |
| Peru | 0.03488 | 0.14147 | -0.04085 | -0.02909 | -0.02334 | -0.02555 |
| Spain | 0.09172 | 0.23378 | -0.01160 | -0.00710 | 0.00214 | -0.00672 |
| Sri Lanka | 0.19403 | 0.27032 | 0.01625 | 0.02550 | 0.02580 | 0.02484 |
| Sweden | 0.05049 | 0.06481 | -0.06583 | -0.04793 | -0.04038 | -0.04620 |
| Switzerland | 0.06763 | 0.04120 | -0.07542 | -0.07212 | -0.06480 | -0.06891 |
| Uganda | 0.28611 | 0.18889 | 0.01732 | 0.01774 | 0.01248 | 0.01562 |
| Uruguay | 0.04787 | 0.14096 | -0.03916 | -0.02283 | -0.01520 | -0.02185 |
| USA 2 | 0.16125 | 0.16170 | -0.02577 | -0.01383 | -0.00640 | -0.01152 |
| USA 3 | 0.26011 | 0.07070 | -0.04069 | -0.02777 | -0.02102 | -0.02583 |

Table 6. Proportion estimates for lifetime abstainers and former drinkers, as well as Population Attributable Fractions (PAF) estimates for pancreatitis using a categorical model and continuous model (Gamma, Log-Normal, and Weibull) for women

|  | Proportions | | PAF Estimates | | | |
| --- | --- | --- | --- | --- | --- | --- |
| Country | Abstainers | Former Drinkers | Categorical | Gamma Model | Log-Normal Model | Weibull Model |
| Argentina | 0.06355 | 0.29933 | 0.12383 | 0.12102 | 0.19875 | 0.12383 |
| Australia | 0.04951 | 0.13319 | 0.07429 | 0.06825 | 0.22384 | 0.07578 |
| Australia 1 | 0.12341 | 0.06414 | 0.04223 | 0.03542 | 0.08875 | 0.03739 |
| Austria | 0.17941 | 0.32880 | 0.15930 | 0.13893 | 0.14866 | 0.14006 |
| Belize | 0.59903 | 0.21449 | 0.11285 | 0.08988 | 0.09982 | 0.09084 |
| Brazil | 0.22741 | 0.36006 | 0.29587 | 0.21202 | 0.31462 | 0.24409 |
| Canada | 0.09532 | 0.16116 | 0.10256 | 0.07857 | 0.14105 | 0.08040 |
| Costa Rica | 0.19253 | 0.37923 | 0.14655 | 0.14461 | 0.16407 | 0.14517 |
| Czech Republic | 0.05538 | 0.14665 | 0.12853 | 0.09043 | 0.24617 | 0.10656 |
| Denmark | 0.00971 | 0.07061 | 0.05214 | 0.04944 | 0.18296 | 0.05037 |
| ECAS: Finland | 0.07480 | 0.00197 | 0.06512 | 0.01121 | 0.03906 | 0.01189 |
| ECAS: France | 0.27238 | 0.00000 | 0.02138 | 0.01473 | 0.11663 | 0.01845 |
| ECAS: Germany | 0.18471 | 0.00000 | 0.05912 | 0.01586 | 0.06885 | 0.01723 |
| ECAS: Italy | 0.21206 | 0.00195 | 0.07671 | 0.03982 | 0.23561 | 0.05287 |
| ECAS: Sweden | 0.13200 | 0.00200 | 0.00823 | 0.00605 | 0.02134 | 0.00615 |
| ECAS: UK | 0.14286 | 0.00000 | 0.16838 | 0.07425 | 0.28355 | 0.09518 |
| Finland | 0.06491 | 0.04057 | 0.04739 | 0.02438 | 0.11709 | 0.02542 |
| France | 0.03552 | 0.41525 | 0.17324 | 0.16547 | 0.17871 | 0.16644 |
| Germany | 0.02588 | 0.03691 | 0.07808 | 0.04452 | 0.19565 | 0.04991 |
| Hungary | 0.17718 | 0.05964 | 0.03276 | 0.02691 | 0.05405 | 0.02711 |
| Iceland | 0.07396 | 0.08261 | 0.04888 | 0.04130 | 0.06996 | 0.04170 |
| India | 0.84014 | 0.10204 | 0.05865 | 0.06494 | 0.09082 | 0.07403 |
| Ireland | 0.23933 | 0.05937 | 0.10040 | 0.07888 | 0.21768 | 0.08503 |
| Isle of Man | 0.01838 | 0.11949 | 0.08637 | 0.08784 | 0.34061 | 0.10871 |
| Israel | 0.42882 | 0.00000 | 0.03666 | 0.00796 | 0.08606 | 0.01343 |
| Italy | 0.19622 | 0.06094 | 0.05988 | 0.05408 | 0.17847 | 0.05821 |
| Japan | 0.15058 | 0.08415 | 0.09380 | 0.04765 | 0.18084 | 0.06491 |
| Kazakhstan | 0.10143 | 0.26307 | 0.11393 | 0.10579 | 0.13977 | 0.10622 |
| Mexico | 0.40553 | 0.17212 | 0.07741 | 0.07110 | 0.07244 | 0.07096 |
| Netherlands | 0.14467 | 0.17495 | 0.09222 | 0.08363 | 0.10507 | 0.08456 |
| Nicaragua | 0.50282 | 0.39336 | 0.19834 | 0.15635 | 0.15740 | 0.15658 |
| Nigeria | 0.56034 | 0.22980 | 0.26630 | 0.26595 | 0.30340 | 0.28019 |
| Norway | 0.04049 | 0.07570 | 0.04408 | 0.03945 | 0.11789 | 0.04038 |
| Peru | 0.08966 | 0.29951 | 0.11923 | 0.11701 | 0.11705 | 0.11705 |
| Spain | 0.22908 | 0.32427 | 0.13699 | 0.13518 | 0.24723 | 0.13888 |
| Sri Lanka | 0.86610 | 0.06949 | 0.02999 | 0.02971 | 0.02972 | 0.02970 |
| Sweden | 0.09666 | 0.11230 | 0.05291 | 0.04994 | 0.05754 | 0.05013 |
| Switzerland | 0.19806 | 0.06082 | 0.05578 | 0.03942 | 0.09945 | 0.04007 |
| Uganda | 0.36412 | 0.26649 | 0.23046 | 0.16673 | 0.26293 | 0.19851 |
| Uruguay | 0.17308 | 0.22596 | 0.13743 | 0.09737 | 0.17224 | 0.10184 |
| USA 1 | 0.10302 | 0.13854 | 0.08059 | 0.06788 | 0.17978 | 0.07739 |
| USA 2 | 0.32630 | 0.18852 | 0.10894 | 0.08263 | 0.10820 | 0.08450 |
| USA 3 | 0.38019 | 0.05805 | 0.05329 | 0.03079 | 0.06957 | 0.03258 |
|  |  |  |  |  |  |  |

Table 7. Proportion estimates for lifetime abstainers and former drinkers, as well as Population Attributable Fraction (PAF) estimates for pancreatitis using a categorical model and a continuous model (Gamma, Log-Normal, and Weibull) for men

|  | Proportions | | PAF Estimates | | | |
| --- | --- | --- | --- | --- | --- | --- |
| Country | Abstainers | Former Drinkers | Categorical | Gamma Model | Log-Normal Model | Weibull Model |
| Argentina | 0.02488 | 0.08209 | 0.22296 | 0.15927 | 0.43723 | 0.20014 |
| Australia | 0.04000 | 0.07800 | 0.08654 | 0.08482 | 0.38073 | 0.10451 |
| Austria | 0.07014 | 0.15774 | 0.28936 | 0.22295 | 0.35679 | 0.23010 |
| Belize | 0.20958 | 0.28647 | 0.33325 | 0.25511 | 0.33768 | 0.27772 |
| Brazil | 0.14516 | 0.27240 | 0.36261 | 0.29194 | 0.39178 | 0.32264 |
| Canada | 0.05019 | 0.13590 | 0.21733 | 0.13400 | 0.33826 | 0.15737 |
| Costa Rica | 0.07212 | 0.24279 | 0.12691 | 0.10615 | 0.29474 | 0.14666 |
| Czech Republic | 0.02653 | 0.07235 | 0.45021 | 0.44383 | 0.59431 | 0.46265 |
| Denmark | 0.00669 | 0.02899 | 0.21317 | 0.12660 | 0.36293 | 0.13557 |
| ECAS: Finland | 0.06855 | 0.00000 | 0.15448 | 0.09867 | 0.28828 | 0.10850 |
| ECAS: France | 0.12632 | 0.00000 | 0.27896 | 0.19846 | 0.44203 | 0.22876 |
| ECAS: Germany | 0.11828 | 0.00000 | 0.11683 | 0.07036 | 0.27456 | 0.07978 |
| ECAS: Italy | 0.10700 | 0.00000 | 0.14476 | 0.13578 | 0.42278 | 0.14220 |
| ECAS: Sweden | 0.07803 | 0.00000 | 0.14909 | 0.04825 | 0.19429 | 0.05417 |
| ECAS: UK | 0.10422 | 0.00000 | 0.52217 | 0.52829 | 0.59284 | 0.54216 |
| Finland | 0.03181 | 0.05196 | 0.12555 | 0.07766 | 0.33705 | 0.08980 |
| France | 0.01975 | 0.20080 | 0.24059 | 0.22953 | 0.39322 | 0.24817 |
| Germany | 0.01415 | 0.03101 | 0.18565 | 0.16192 | 0.44023 | 0.17239 |
| Hungary | 0.04696 | 0.04052 | 0.08571 | 0.05040 | 0.29853 | 0.07345 |
| Iceland | 0.04117 | 0.09005 | 0.05270 | 0.04367 | 0.15151 | 0.04482 |
| India | 0.56138 | 0.10816 | 0.40834 | 0.37193 | 0.37013 | 0.36623 |
| Ireland | 0.16501 | 0.06958 | 0.58943 | 0.51065 | 0.57120 | 0.52348 |
| Isle of Man | 0.00885 | 0.06195 | 0.41981 | 0.39645 | 0.57738 | 0.43202 |
| Israel | 0.23209 | 0.00000 | 0.15418 | 0.05803 | 0.26452 | 0.09695 |
| Italy | 0.05808 | 0.03977 | 0.13931 | 0.18626 | 0.45169 | 0.18221 |
| Japan | 0.04869 | 0.04148 | 0.25401 | 0.27050 | 0.53622 | 0.35619 |
| Kazakhstan | 0.04267 | 0.21336 | 0.42465 | 0.28435 | 0.44050 | 0.31483 |
| Mexico | 0.09404 | 0.13644 | 0.29837 | 0.18655 | 0.36514 | 0.24155 |
| Netherlands | 0.06032 | 0.10269 | 0.13115 | 0.11909 | 0.29457 | 0.12463 |
| Nicaragua | 0.12052 | 0.45114 | 0.35959 | 0.24862 | 0.30616 | 0.26612 |
| Nigeria | 0.41863 | 0.18445 | 0.35308 | 0.38344 | 0.43277 | 0.39344 |
| Norway | 0.02321 | 0.06286 | 0.13943 | 0.06723 | 0.26520 | 0.07835 |
| Peru | 0.03488 | 0.14147 | 0.17245 | 0.04226 | 0.05929 | 0.04328 |
| Spain | 0.09172 | 0.23378 | 0.22602 | 0.19353 | 0.43043 | 0.20917 |
| Sri Lanka | 0.19403 | 0.27032 | 0.36577 | 0.32401 | 0.36493 | 0.33343 |
| Sweden | 0.05049 | 0.06481 | 0.03966 | 0.02675 | 0.08574 | 0.02787 |
| Switzerland | 0.06763 | 0.04120 | 0.25281 | 0.12645 | 0.30119 | 0.14034 |
| Uganda | 0.28611 | 0.18889 | 0.56306 | 0.55651 | 0.55602 | 0.55421 |
| Uruguay | 0.04787 | 0.14096 | 0.39759 | 0.24541 | 0.43675 | 0.29331 |
| USA 2 | 0.16125 | 0.16170 | 0.18083 | 0.11673 | 0.28693 | 0.15662 |
| USA 3 | 0.26011 | 0.07070 | 0.24730 | 0.12414 | 0.28998 | 0.16304 |

**Table 8. Count estimates for drinkers that drink ≤ 96 g/day, > 96 g/day, ≤ 120 g/day, and > 120 g/day, as well as proportion estimates for male drinkers that report drinking above 96 g/day and 120 g/day. The weighted global proportion estimates for male drinkers that report drinking above 96 g/day and 120 g/day are 3.90% and 2.73%, respectively.**

|  | Counts | | | | | Proportion of Heavy Drinkers | |
| --- | --- | --- | --- | --- | --- | --- | --- |
| Country | ≤ 96 g/day | > 96 g/day | ≤ 120 g/day | > 120 g/day | Total | > 96 g/day | > 120 g/day |
| Argentina | 355 | 4 | 356 | 3 | 359 | 0.01114 | 0.00836 |
| Australia | 881 | 1 | 881 | 1 | 882 | 0.00113 | 0.00113 |
| Austria | 2631 | 66 | 2672 | 25 | 2697 | 0.02447 | 0.00927 |
| Belize | 921 | 36 | 930 | 27 | 957 | 0.03762 | 0.02821 |
| Brazil | 310 | 15 | 315 | 10 | 325 | 0.04615 | 0.03077 |
| Canada | 4767 | 66 | 4791 | 42 | 4833 | 0.01366 | 0.00869 |
| Costa Rica | 282 | 3 | 284 | 1 | 285 | 0.01053 | 0.00351 |
| Czech Republic | 1061 | 60 | 1089 | 32 | 1121 | 0.05352 | 0.02855 |
| Denmark | 853 | 12 | 858 | 7 | 865 | 0.01387 | 0.00809 |
| ECAS: Finland | 459 | 3 | 460 | 2 | 462 | 0.00649 | 0.00433 |
| ECAS: France | 407 | 8 | 411 | 4 | 415 | 0.01928 | 0.00964 |
| ECAS: Germany | 327 | 1 | 327 | 1 | 328 | 0.00305 | 0.00305 |
| ECAS: Italy | 431 | 3 | 432 | 2 | 434 | 0.00691 | 0.00461 |
| ECAS: Sweden | 446 | 3 | 447 | 2 | 449 | 0.00668 | 0.00445 |
| ECAS: UK | 344 | 17 | 348 | 13 | 361 | 0.04709 | 0.03601 |
| Finland | 860 | 4 | 862 | 2 | 864 | 0.00463 | 0.00231 |
| France | 4625 | 72 | 4661 | 36 | 4697 | 0.01533 | 0.00766 |
| Germany | 3468 | 42 | 3495 | 15 | 3510 | 0.01197 | 0.00427 |
| Hungary | 988 | 3 | 990 | 1 | 991 | 0.00303 | 0.00101 |
| Iceland | 1012 | 1 | 1013 | 0 | 1013 | 0.00099 | 0.00000 |
| India | 447 | 51 | 462 | 36 | 498 | 0.10241 | 0.07229 |
| Ireland | 353 | 32 | 361 | 24 | 385 | 0.08312 | 0.06234 |
| Isle of Man | 403 | 17 | 409 | 11 | 420 | 0.04048 | 0.02619 |
| Israel | 1984 | 21 | 1991 | 14 | 2005 | 0.01047 | 0.00698 |
| Italy | 1427 | 2 | 1429 | 0 | 1429 | 0.00140 | 0.00000 |
| Japan | 1001 | 8 | 1001 | 8 | 1009 | 0.00793 | 0.00793 |
| Kazakhstan | 385 | 16 | 388 | 13 | 401 | 0.03990 | 0.03242 |
| Mexico | 1785 | 48 | 1801 | 32 | 1833 | 0.02619 | 0.01746 |
| Netherlands | 1665 | 14 | 1671 | 8 | 1679 | 0.00834 | 0.00476 |
| Nicaragua | 248 | 15 | 252 | 11 | 263 | 0.05703 | 0.04183 |
| Nigeria | 408 | 31 | 421 | 18 | 439 | 0.07062 | 0.04100 |
| Norway | 937 | 8 | 941 | 4 | 945 | 0.00847 | 0.00423 |
| Peru | 421 | 4 | 421 | 4 | 425 | 0.00941 | 0.00941 |
| Spain | 593 | 10 | 599 | 4 | 603 | 0.01658 | 0.00663 |
| Sri Lanka | 299 | 24 | 304 | 19 | 323 | 0.07430 | 0.05882 |
| Sweden | 2343 | 5 | 2348 | 0 | 2348 | 0.00213 | 0.00000 |
| Switzerland | 5046 | 80 | 5077 | 49 | 5126 | 0.01561 | 0.00956 |
| Uganda | 327 | 51 | 346 | 32 | 378 | 0.13492 | 0.08466 |
| Uruguay | 293 | 12 | 297 | 8 | 305 | 0.03934 | 0.02623 |
| USA 2 | 1476 | 23 | 1486 | 13 | 1499 | 0.01534 | 0.00867 |
| USA 3 | 2250 | 50 | 2267 | 33 | 2300 | 0.02174 | 0.01435 |

**Table 9. Count estimates for drinkers that drink ≤ 96 g/day, > 96 g/day, ≤ 120 g/day, and > 120 g/day, as well as proportion estimates for female drinkers that report drinking above 96 g/day and 120 g/day. The weighted global proportion estimates for female drinkers that report drinking above 96 g/day and 120 g/day are 1.61% and 1.12%, respectively.**

|  | Counts | | | | | Proportion of Heavy Drinkers | |
| --- | --- | --- | --- | --- | --- | --- | --- |
| Country | ≤ 96 g/day | > 96 g/day | ≤ 120 g/day | > 120 g/day | Total | > 96 g/day | > 120 g/day |
| Argentina | 381 | 0 | 381 | 0 | 381 | 0.00000 | 0.00000 |
| Australia | 1170 | 2 | 1172 | 0 | 1172 | 0.00171 | 0.00000 |
| Australia 1 | 3002 | 0 | 3002 | 0 | 3002 | 0.00000 | 0.00000 |
| Austria | 1908 | 8 | 1911 | 5 | 1916 | 0.00418 | 0.00261 |
| Belize | 381 | 5 | 382 | 4 | 386 | 0.01295 | 0.01036 |
| Brazil | 273 | 10 | 275 | 8 | 283 | 0.03534 | 0.02827 |
| Canada | 5840 | 10 | 5843 | 7 | 5850 | 0.00171 | 0.00120 |
| Costa Rica | 367 | 0 | 367 | 0 | 367 | 0.00000 | 0.00000 |
| Czech Republic | 1018 | 5 | 1021 | 2 | 1023 | 0.00489 | 0.00196 |
| Denmark | 1042 | 0 | 1042 | 0 | 1042 | 0.00000 | 0.00000 |
| ECAS: Finland | 468 | 1 | 468 | 1 | 469 | 0.00213 | 0.00213 |
| ECAS: France | 382 | 0 | 382 | 0 | 382 | 0.00000 | 0.00000 |
| ECAS: Germany | 511 | 1 | 511 | 1 | 512 | 0.00195 | 0.00195 |
| ECAS: Italy | 402 | 2 | 402 | 2 | 404 | 0.00495 | 0.00495 |
| ECAS: Sweden | 433 | 0 | 433 | 0 | 433 | 0.00000 | 0.00000 |
| ECAS: UK | 493 | 5 | 494 | 4 | 498 | 0.01004 | 0.00803 |
| Finland | 880 | 2 | 881 | 1 | 882 | 0.00227 | 0.00113 |
| France | 4200 | 6 | 4204 | 2 | 4206 | 0.00143 | 0.00048 |
| Germany | 4152 | 12 | 4160 | 4 | 4164 | 0.00288 | 0.00096 |
| Hungary | 883 | 0 | 883 | 0 | 883 | 0.00000 | 0.00000 |
| Iceland | 1072 | 0 | 1072 | 0 | 1072 | 0.00000 | 0.00000 |
| India | 83 | 2 | 85 | 0 | 85 | 0.02353 | 0.00000 |
| Ireland | 374 | 4 | 377 | 1 | 378 | 0.01058 | 0.00265 |
| Isle of Man | 469 | 0 | 469 | 0 | 469 | 0.00000 | 0.00000 |
| Israel | 1930 | 8 | 1935 | 3 | 1938 | 0.00413 | 0.00155 |
| Italy | 1219 | 0 | 1219 | 0 | 1219 | 0.00000 | 0.00000 |
| Japan | 862 | 2 | 862 | 2 | 864 | 0.00231 | 0.00231 |
| Kazakhstan | 401 | 0 | 401 | 0 | 401 | 0.00000 | 0.00000 |
| Mexico | 1404 | 2 | 1405 | 1 | 1406 | 0.00142 | 0.00071 |
| Netherlands | 1504 | 1 | 1504 | 1 | 1505 | 0.00066 | 0.00066 |
| Nicaragua | 142 | 5 | 143 | 4 | 147 | 0.03401 | 0.02721 |
| Nigeria | 186 | 14 | 192 | 8 | 200 | 0.07000 | 0.04000 |
| Norway | 1004 | 0 | 1004 | 0 | 1004 | 0.00000 | 0.00000 |
| Peru | 620 | 0 | 620 | 0 | 620 | 0.00000 | 0.00000 |
| Spain | 427 | 0 | 427 | 0 | 427 | 0.00000 | 0.00000 |
| Sri Lanka | 38 | 0 | 38 | 0 | 38 | 0.00000 | 0.00000 |
| Sweden | 2226 | 0 | 2226 | 0 | 2226 | 0.00000 | 0.00000 |
| Switzerland | 5356 | 6 | 5357 | 5 | 5362 | 0.00112 | 0.00093 |
| Uganda | 272 | 8 | 275 | 5 | 280 | 0.02857 | 0.01786 |
| Uruguay | 374 | 1 | 374 | 1 | 375 | 0.00267 | 0.00267 |
| USA 1 | 852 | 2 | 852 | 2 | 854 | 0.00234 | 0.00234 |
| USA 2 | 1306 | 4 | 1307 | 3 | 1310 | 0.00305 | 0.00229 |
| USA 3 | 2268 | 6 | 2271 | 3 | 2274 | 0.00264 | 0.00132 |

Table 10. Proportion estimates for the decomposition of alcohol Population Attributable Fraction (PAF) for breast cancer consisting of drinkers that drink ≤ 96 g/day and > 96 g/day using a continuous model (Gamma, Log-Normal, and Weibull) for women

|  | Gamma Model | | Log Normal Model | | Weibull Model | |
| --- | --- | --- | --- | --- | --- | --- |
| Country | ≤ 96 g/day | > 96 g/day | ≤ 96 g/day | > 96 g/day | ≤ 96 g/day | > 96 g/day |
| Argentina | 0.999971 | 0.000029 | 0.893719 | 0.106281 | 0.997853 | 0.002147 |
| Australia | 0.999312 | 0.000688 | 0.739494 | 0.260506 | 0.989016 | 0.010984 |
| Australia 1 | 0.999951 | 0.000049 | 0.865949 | 0.134051 | 0.997605 | 0.002395 |
| Austria | 0.999908 | 0.000092 | 0.988866 | 0.011134 | 0.999780 | 0.000220 |
| Belize | 0.999543 | 0.000457 | 0.977667 | 0.022333 | 0.997073 | 0.002927 |
| Brazil | 0.937348 | 0.062652 | 0.784340 | 0.215660 | 0.889187 | 0.110813 |
| Canada | 0.999775 | 0.000225 | 0.900060 | 0.099940 | 0.998167 | 0.001833 |
| Costa Rica | 1.000000 | 0.000000 | 0.975587 | 0.024413 | 0.999704 | 0.000296 |
| Czech Republic | 0.992434 | 0.007566 | 0.746082 | 0.253918 | 0.967252 | 0.032748 |
| Denmark | 0.999692 | 0.000308 | 0.791498 | 0.208502 | 0.999188 | 0.000812 |
| ECAS: Finland | 1.000000 | 0.000000 | 0.925321 | 0.074679 | 0.999894 | 0.000106 |
| ECAS: France | 0.998903 | 0.001097 | 0.705689 | 0.294311 | 0.990033 | 0.009967 |
| ECAS: Germany | 0.999712 | 0.000288 | 0.839467 | 0.160533 | 0.997337 | 0.002663 |
| ECAS: Italy | 0.985955 | 0.014045 | 0.599199 | 0.400801 | 0.957107 | 0.042893 |
| ECAS: Sweden | 1.000000 | 0.000000 | 0.956256 | 0.043744 | 1.000000 | 0.000000 |
| ECAS: UK | 0.962960 | 0.037040 | 0.602993 | 0.397007 | 0.924522 | 0.075478 |
| Finland | 1.000000 | 0.000000 | 0.778385 | 0.221615 | 0.999509 | 0.000491 |
| France | 0.999888 | 0.000112 | 0.985732 | 0.014268 | 0.999600 | 0.000400 |
| Germany | 0.997191 | 0.002809 | 0.731543 | 0.268457 | 0.988566 | 0.011434 |
| Hungary | 1.000000 | 0.000000 | 0.901718 | 0.098282 | 0.999946 | 0.000054 |
| Iceland | 1.000000 | 0.000000 | 0.943719 | 0.056281 | 0.999985 | 0.000015 |
| India | 0.939348 | 0.060652 | 0.835070 | 0.164930 | 0.902046 | 0.097954 |
| Ireland | 0.984504 | 0.015496 | 0.759578 | 0.240422 | 0.975023 | 0.024977 |
| Isle of Man | 0.989171 | 0.010829 | 0.620627 | 0.379373 | 0.959428 | 0.040572 |
| Israel | 0.998045 | 0.001955 | 0.612081 | 0.387919 | 0.959108 | 0.040892 |
| Italy | 0.996270 | 0.003730 | 0.771986 | 0.228014 | 0.989855 | 0.010145 |
| Japan | 0.997641 | 0.002359 | 0.696182 | 0.303818 | 0.953418 | 0.046582 |
| Kazakhstan | 1.000000 | 0.000000 | 0.948100 | 0.051900 | 0.999697 | 0.000303 |
| Mexico | 1.000000 | 0.000000 | 0.996793 | 0.003207 | 0.999973 | 0.000027 |
| Netherlands | 0.999938 | 0.000062 | 0.968597 | 0.031403 | 0.999690 | 0.000310 |
| Nicaragua | 0.993690 | 0.006310 | 0.988826 | 0.011174 | 0.991629 | 0.008371 |
| Nigeria | 0.767172 | 0.232828 | 0.671068 | 0.328932 | 0.731585 | 0.268415 |
| Norway | 1.000000 | 0.000000 | 0.839931 | 0.160069 | 0.999714 | 0.000286 |
| Peru | 1.000000 | 0.000000 | 0.999992 | 0.000008 | 1.000000 | 0.000000 |
| Spain | 0.999106 | 0.000894 | 0.850651 | 0.149349 | 0.995287 | 0.004713 |
| Sri Lanka | 1.000000 | 0.000000 | 0.999866 | 0.000134 | 1.000000 | 0.000000 |
| Sweden | 1.000000 | 0.000000 | 0.986436 | 0.013564 | 1.000000 | 0.000000 |
| Switzerland | 0.999918 | 0.000082 | 0.886065 | 0.113935 | 0.999755 | 0.000245 |
| Uganda | 0.940846 | 0.059154 | 0.777391 | 0.222609 | 0.885812 | 0.114188 |
| Uruguay | 0.999721 | 0.000279 | 0.877691 | 0.122309 | 0.993457 | 0.006543 |
| USA 1 | 0.999223 | 0.000777 | 0.789041 | 0.210959 | 0.982602 | 0.017398 |
| USA 2 | 0.999733 | 0.000267 | 0.947773 | 0.052227 | 0.995838 | 0.004162 |
| USA 3 | 0.999835 | 0.000165 | 0.867239 | 0.132761 | 0.994662 | 0.005338 |

Table 11. Proportion estimates for the decomposition of alcohol Population Attributable Fraction (PAF) for breast cancer consisting of drinkers that drink ≤ 120 g/day and > 120 g/day using a continuous model (Gamma, Log-Normal, and Weibull) for women

|  | Gamma Model | | Log Normal Model | | Weibull Model | |
| --- | --- | --- | --- | --- | --- | --- |
| Country | ≤ 120 g/day | > 120 g/day | ≤ 120 g/day | > 120 g/day | ≤ 120 g/day | > 120 g/day |
| Argentina | 1.000000 | 0.000000 | 0.909659 | 0.090341 | 0.999073 | 0.000927 |
| Australia | 0.999885 | 0.000115 | 0.777368 | 0.222632 | 0.994921 | 0.005079 |
| Australia 1 | 1.000000 | 0.000000 | 0.892246 | 0.107754 | 0.999240 | 0.000760 |
| Austria | 0.999994 | 0.000006 | 0.993113 | 0.006887 | 0.999976 | 0.000024 |
| Belize | 0.999907 | 0.000093 | 0.982202 | 0.017798 | 0.998584 | 0.001416 |
| Brazil | 0.961079 | 0.038921 | 0.810008 | 0.189992 | 0.913560 | 0.086440 |
| Canada | 0.999972 | 0.000028 | 0.920868 | 0.079132 | 0.999492 | 0.000508 |
| Costa Rica | 1.000000 | 0.000000 | 0.979850 | 0.020150 | 0.999888 | 0.000112 |
| Czech Republic | 0.997785 | 0.002215 | 0.785377 | 0.214623 | 0.982347 | 0.017653 |
| Denmark | 0.999967 | 0.000033 | 0.832468 | 0.167532 | 0.999868 | 0.000132 |
| ECAS: Finland | 1.000000 | 0.000000 | 0.946542 | 0.053458 | 1.000000 | 0.000000 |
| ECAS: France | 0.999846 | 0.000154 | 0.758912 | 0.241088 | 0.996531 | 0.003469 |
| ECAS: Germany | 0.999979 | 0.000021 | 0.876285 | 0.123715 | 0.999345 | 0.000655 |
| ECAS: Italy | 0.996021 | 0.003979 | 0.663270 | 0.336730 | 0.980119 | 0.019881 |
| ECAS: Sweden | 1.000000 | 0.000000 | 0.969927 | 0.030073 | 1.000000 | 0.000000 |
| ECAS: UK | 0.986469 | 0.013531 | 0.669172 | 0.330828 | 0.961513 | 0.038487 |
| Finland | 1.000000 | 0.000000 | 0.817450 | 0.182550 | 0.999902 | 0.000098 |
| France | 0.999989 | 0.000011 | 0.990305 | 0.009695 | 0.999931 | 0.000069 |
| Germany | 0.999473 | 0.000527 | 0.780900 | 0.219100 | 0.996047 | 0.003953 |
| Hungary | 1.000000 | 0.000000 | 0.919124 | 0.080876 | 1.000000 | 0.000000 |
| Iceland | 1.000000 | 0.000000 | 0.958031 | 0.041969 | 1.000000 | 0.000000 |
| India | 0.958957 | 0.041043 | 0.853620 | 0.146380 | 0.921128 | 0.078872 |
| Ireland | 0.995524 | 0.004476 | 0.809483 | 0.190517 | 0.991054 | 0.008946 |
| Isle of Man | 0.996669 | 0.003331 | 0.672039 | 0.327961 | 0.978479 | 0.021521 |
| Israel | 0.999631 | 0.000369 | 0.669737 | 0.330263 | 0.977054 | 0.022946 |
| Italy | 0.999223 | 0.000777 | 0.816106 | 0.183894 | 0.996725 | 0.003275 |
| Japan | 0.999470 | 0.000530 | 0.736585 | 0.263415 | 0.969840 | 0.030160 |
| Kazakhstan | 1.000000 | 0.000000 | 0.956647 | 0.043353 | 0.999896 | 0.000104 |
| Mexico | 1.000000 | 0.000000 | 0.997571 | 0.002429 | 1.000000 | 0.000000 |
| Netherlands | 0.999991 | 0.000009 | 0.978041 | 0.021959 | 0.999956 | 0.000044 |
| Nicaragua | 0.996855 | 0.003145 | 0.991012 | 0.008988 | 0.994374 | 0.005626 |
| Nigeria | 0.814438 | 0.185562 | 0.703888 | 0.296112 | 0.771883 | 0.228117 |
| Norway | 1.000000 | 0.000000 | 0.870072 | 0.129928 | 0.999940 | 0.000060 |
| Peru | 1.000000 | 0.000000 | 1.000000 | 0.000000 | 1.000000 | 0.000000 |
| Spain | 0.999815 | 0.000185 | 0.872202 | 0.127798 | 0.998033 | 0.001967 |
| Sri Lanka | 1.000000 | 0.000000 | 0.999900 | 0.000100 | 1.000000 | 0.000000 |
| Sweden | 1.000000 | 0.000000 | 0.990495 | 0.009505 | 1.000000 | 0.000000 |
| Switzerland | 1.000000 | 0.000000 | 0.913529 | 0.086471 | 0.999973 | 0.000027 |
| Uganda | 0.964458 | 0.035542 | 0.804406 | 0.195594 | 0.911313 | 0.088687 |
| Uruguay | 0.999956 | 0.000044 | 0.896292 | 0.103708 | 0.996663 | 0.003337 |
| USA 1 | 0.999858 | 0.000142 | 0.819867 | 0.180133 | 0.990354 | 0.009646 |
| USA 2 | 0.999959 | 0.000041 | 0.957762 | 0.042238 | 0.998050 | 0.001950 |
| USA 3 | 0.999979 | 0.000021 | 0.891633 | 0.108367 | 0.997758 | 0.002242 |

Table 12. Proportion estimates for the decomposition of alcohol Population Attributable Fraction (PAF) for breast cancer consisting of drinkers that drink ≤ 150 g/day and > 150 g/day using a continuous model (Gamma, Log-Normal, and Weibull) for women

|  | Gamma Model | | Log Normal Model | | Weibull Model | |
| --- | --- | --- | --- | --- | --- | --- |
| Country | ≤ 150 g/day | > 150 g/day | ≤ 150 g/day | > 150 g/day | ≤ 150 g/day | > 150 g/day |
| Argentina | 1.000000 | 0.000000 | 0.926980 | 0.073020 | 0.999649 | 0.000351 |
| Australia | 0.999990 | 0.000010 | 0.819091 | 0.180909 | 0.997946 | 0.002054 |
| Australia 1 | 1.000000 | 0.000000 | 0.917745 | 0.082255 | 0.999802 | 0.000198 |
| Austria | 1.000000 | 0.000000 | 0.995968 | 0.004032 | 1.000000 | 0.000000 |
| Belize | 0.999990 | 0.000010 | 0.986540 | 0.013460 | 0.999402 | 0.000598 |
| Brazil | 0.978934 | 0.021066 | 0.840573 | 0.159427 | 0.937087 | 0.062913 |
| Canada | 1.000000 | 0.000000 | 0.940581 | 0.059419 | 0.999897 | 0.000103 |
| Costa Rica | 1.000000 | 0.000000 | 0.984196 | 0.015804 | 0.999960 | 0.000040 |
| Czech Republic | 0.999532 | 0.000468 | 0.827677 | 0.172323 | 0.991558 | 0.008442 |
| Denmark | 1.000000 | 0.000000 | 0.872401 | 0.127599 | 0.999989 | 0.000011 |
| ECAS: Finland | 1.000000 | 0.000000 | 0.963662 | 0.036338 | 1.000000 | 0.000000 |
| ECAS: France | 0.999978 | 0.000022 | 0.812565 | 0.187435 | 0.999012 | 0.000988 |
| ECAS: Germany | 1.000000 | 0.000000 | 0.909582 | 0.090418 | 0.999873 | 0.000127 |
| ECAS: Italy | 0.999199 | 0.000801 | 0.731459 | 0.268541 | 0.992153 | 0.007847 |
| ECAS: Sweden | 1.000000 | 0.000000 | 0.980319 | 0.019681 | 1.000000 | 0.000000 |
| ECAS: UK | 0.996246 | 0.003754 | 0.738558 | 0.261442 | 0.983131 | 0.016869 |
| Finland | 1.000000 | 0.000000 | 0.857177 | 0.142823 | 0.999980 | 0.000020 |
| France | 1.000000 | 0.000000 | 0.993759 | 0.006241 | 0.999995 | 0.000005 |
| Germany | 0.999931 | 0.000069 | 0.830417 | 0.169583 | 0.998908 | 0.001092 |
| Hungary | 1.000000 | 0.000000 | 0.936707 | 0.063293 | 1.000000 | 0.000000 |
| Iceland | 1.000000 | 0.000000 | 0.970302 | 0.029698 | 1.000000 | 0.000000 |
| India | 0.975356 | 0.024644 | 0.876194 | 0.123806 | 0.940574 | 0.059426 |
| Ireland | 0.999085 | 0.000915 | 0.857202 | 0.142798 | 0.997538 | 0.002462 |
| Isle of Man | 0.999257 | 0.000743 | 0.730381 | 0.269619 | 0.989959 | 0.010041 |
| Israel | 0.999963 | 0.000037 | 0.732557 | 0.267443 | 0.988265 | 0.011735 |
| Italy | 0.999900 | 0.000100 | 0.859450 | 0.140550 | 0.999181 | 0.000819 |
| Japan | 0.999916 | 0.000084 | 0.782583 | 0.217417 | 0.981957 | 0.018043 |
| Kazakhstan | 1.000000 | 0.000000 | 0.965567 | 0.034433 | 0.999965 | 0.000035 |
| Mexico | 1.000000 | 0.000000 | 0.998242 | 0.001758 | 1.000000 | 0.000000 |
| Netherlands | 1.000000 | 0.000000 | 0.985447 | 0.014553 | 1.000000 | 0.000000 |
| Nicaragua | 0.998698 | 0.001302 | 0.993147 | 0.006853 | 0.996519 | 0.003481 |
| Nigeria | 0.864116 | 0.135884 | 0.745596 | 0.254404 | 0.817570 | 0.182430 |
| Norway | 1.000000 | 0.000000 | 0.899876 | 0.100124 | 1.000000 | 0.000000 |
| Peru | 1.000000 | 0.000000 | 1.000000 | 0.000000 | 1.000000 | 0.000000 |
| Spain | 0.999974 | 0.000026 | 0.896058 | 0.103942 | 0.999307 | 0.000693 |
| Sri Lanka | 1.000000 | 0.000000 | 0.999933 | 0.000067 | 1.000000 | 0.000000 |
| Sweden | 1.000000 | 0.000000 | 0.993668 | 0.006332 | 1.000000 | 0.000000 |
| Switzerland | 1.000000 | 0.000000 | 0.937821 | 0.062179 | 1.000000 | 0.000000 |
| Uganda | 0.981526 | 0.018474 | 0.836335 | 0.163665 | 0.935719 | 0.064281 |
| Uruguay | 1.000000 | 0.000000 | 0.916388 | 0.083612 | 0.998481 | 0.001519 |
| USA 1 | 0.999989 | 0.000011 | 0.853714 | 0.146286 | 0.995150 | 0.004850 |
| USA 2 | 1.000000 | 0.000000 | 0.967556 | 0.032444 | 0.999193 | 0.000807 |
| USA 3 | 1.000000 | 0.000000 | 0.915970 | 0.084030 | 0.999189 | 0.000811 |

Table 13. Proportion estimates for the decomposition of alcohol Population Attributable Fraction (PAF) for breast cancer consisting of drinkers that drink ≤ 200 g/day and > 200 g/day using a continuous model (Gamma, Log-Normal, and Weibull) for women

|  | Gamma Model | | Log Normal Model | | Weibull Model | |
| --- | --- | --- | --- | --- | --- | --- |
| Country | ≤ 200 g/day | > 200 g/day | ≤ 200 g/day | > 200 g/day | ≤ 200 g/day | > 200 g/day |
| Argentina | 1.000000 | 0.000000 | 0.952436 | 0.047564 | 0.999927 | 0.000073 |
| Australia | 1.000000 | 0.000000 | 0.881348 | 0.118652 | 0.999504 | 0.000496 |
| Australia 1 | 1.000000 | 0.000000 | 0.950385 | 0.049615 | 0.999967 | 0.000033 |
| Austria | 1.000000 | 0.000000 | 0.998272 | 0.001728 | 1.000000 | 0.000000 |
| Belize | 1.000000 | 0.000000 | 0.991982 | 0.008018 | 0.999843 | 0.000157 |
| Brazil | 0.992912 | 0.007088 | 0.890662 | 0.109338 | 0.965306 | 0.034694 |
| Canada | 1.000000 | 0.000000 | 0.964955 | 0.035045 | 0.999991 | 0.000009 |
| Costa Rica | 1.000000 | 0.000000 | 0.990101 | 0.009899 | 0.999993 | 0.000007 |
| Czech Republic | 0.999967 | 0.000033 | 0.888903 | 0.111097 | 0.997459 | 0.002541 |
| Denmark | 1.000000 | 0.000000 | 0.923409 | 0.076591 | 1.000000 | 0.000000 |
| ECAS: Finland | 1.000000 | 0.000000 | 0.981122 | 0.018878 | 1.000000 | 0.000000 |
| ECAS: France | 1.000000 | 0.000000 | 0.884287 | 0.115713 | 0.999868 | 0.000132 |
| ECAS: Germany | 1.000000 | 0.000000 | 0.948514 | 0.051486 | 1.000000 | 0.000000 |
| ECAS: Italy | 0.999947 | 0.000053 | 0.828581 | 0.171419 | 0.998256 | 0.001744 |
| ECAS: Sweden | 1.000000 | 0.000000 | 0.990235 | 0.009765 | 1.000000 | 0.000000 |
| ECAS: UK | 0.999575 | 0.000425 | 0.835310 | 0.164690 | 0.995682 | 0.004318 |
| Finland | 1.000000 | 0.000000 | 0.910994 | 0.089006 | 1.000000 | 0.000000 |
| France | 1.000000 | 0.000000 | 0.996978 | 0.003022 | 1.000000 | 0.000000 |
| Germany | 1.000000 | 0.000000 | 0.896008 | 0.103992 | 0.999862 | 0.000138 |
| Hungary | 1.000000 | 0.000000 | 0.960487 | 0.039513 | 1.000000 | 0.000000 |
| Iceland | 1.000000 | 0.000000 | 0.983753 | 0.016247 | 1.000000 | 0.000000 |
| India | 0.990260 | 0.009740 | 0.914124 | 0.085876 | 0.965616 | 0.034384 |
| Ireland | 0.999938 | 0.000062 | 0.916318 | 0.083682 | 0.999717 | 0.000283 |
| Isle of Man | 0.999942 | 0.000058 | 0.820429 | 0.179571 | 0.997103 | 0.002897 |
| Israel | 1.000000 | 0.000000 | 0.825322 | 0.174678 | 0.996008 | 0.003992 |
| Italy | 1.000000 | 0.000000 | 0.915299 | 0.084701 | 0.999911 | 0.000089 |
| Japan | 1.000000 | 0.000000 | 0.854182 | 0.145818 | 0.992327 | 0.007673 |
| Kazakhstan | 1.000000 | 0.000000 | 0.978077 | 0.021923 | 0.999991 | 0.000009 |
| Mexico | 1.000000 | 0.000000 | 0.999007 | 0.000993 | 1.000000 | 0.000000 |
| Netherlands | 1.000000 | 0.000000 | 0.992689 | 0.007311 | 1.000000 | 0.000000 |
| Nicaragua | 0.999713 | 0.000287 | 0.995874 | 0.004126 | 0.998469 | 0.001531 |
| Nigeria | 0.926864 | 0.073136 | 0.819479 | 0.180521 | 0.884999 | 0.115001 |
| Norway | 1.000000 | 0.000000 | 0.938860 | 0.061140 | 1.000000 | 0.000000 |
| Peru | 1.000000 | 0.000000 | 1.000000 | 0.000000 | 1.000000 | 0.000000 |
| Spain | 1.000000 | 0.000000 | 0.931796 | 0.068204 | 0.999868 | 0.000132 |
| Sri Lanka | 1.000000 | 0.000000 | 0.999967 | 0.000033 | 1.000000 | 0.000000 |
| Sweden | 1.000000 | 0.000000 | 0.996784 | 0.003216 | 1.000000 | 0.000000 |
| Switzerland | 1.000000 | 0.000000 | 0.965365 | 0.034635 | 1.000000 | 0.000000 |
| Uganda | 0.994166 | 0.005834 | 0.888195 | 0.111805 | 0.964721 | 0.035279 |
| Uruguay | 1.000000 | 0.000000 | 0.945729 | 0.054271 | 0.999561 | 0.000439 |
| USA 1 | 1.000000 | 0.000000 | 0.904098 | 0.095902 | 0.998391 | 0.001609 |
| USA 2 | 1.000000 | 0.000000 | 0.980258 | 0.019742 | 0.999801 | 0.000199 |
| USA 3 | 1.000000 | 0.000000 | 0.948236 | 0.051764 | 0.999829 | 0.000171 |

Table 14. Proportion estimates for the decomposition of alcohol Population Attributable Fraction (PAF) for pancreatitis consisting of drinkers that drink ≤ 96 g/day and > 96 g/day using a continuous model (Gamma, Log-Normal, and Weibull) for women

|  | Gamma Model | | Log Normal Model | | Weibull Model | |
| --- | --- | --- | --- | --- | --- | --- |
| Country | ≤ 96 g/day | > 96 g/day | ≤ 96 g/day | > 96 g/day | ≤ 96 g/day | > 96 g/day |
| Argentina | 0.999909 | 0.000091 | 0.602812 | 0.397188 | 0.989050 | 0.010950 |
| Australia | 0.996699 | 0.003301 | 0.314035 | 0.685965 | 0.933081 | 0.066919 |
| Australia 1 | 0.999720 | 0.000280 | 0.461334 | 0.538666 | 0.983952 | 0.016048 |
| Austria | 0.999662 | 0.000338 | 0.936714 | 0.063286 | 0.999179 | 0.000821 |
| Belize | 0.998267 | 0.001733 | 0.889039 | 0.110961 | 0.985312 | 0.014688 |
| Brazil | 0.725260 | 0.274740 | 0.409210 | 0.590790 | 0.585810 | 0.414190 |
| Canada | 0.999034 | 0.000966 | 0.577259 | 0.422741 | 0.990357 | 0.009643 |
| Costa Rica | 1.000000 | 0.000000 | 0.881753 | 0.118247 | 0.998651 | 0.001349 |
| Czech Republic | 0.959573 | 0.040427 | 0.318348 | 0.681652 | 0.815768 | 0.184232 |
| Denmark | 0.998221 | 0.001779 | 0.330948 | 0.669052 | 0.994919 | 0.005081 |
| ECAS: Finland | 0.999911 | 0.000089 | 0.465153 | 0.534847 | 0.998824 | 0.001176 |
| ECAS: France | 0.988761 | 0.011239 | 0.199702 | 0.800298 | 0.894604 | 0.105396 |
| ECAS: Germany | 0.996866 | 0.003134 | 0.306571 | 0.693429 | 0.968526 | 0.031474 |
| ECAS: Italy | 0.894136 | 0.105864 | 0.157807 | 0.842193 | 0.705557 | 0.294443 |
| ECAS: Sweden | 1.000000 | 0.000000 | 0.567599 | 0.432401 | 1.000000 | 0.000000 |
| ECAS: UK | 0.772425 | 0.227575 | 0.162487 | 0.837513 | 0.593122 | 0.406878 |
| Finland | 0.999959 | 0.000041 | 0.305482 | 0.694518 | 0.996433 | 0.003567 |
| France | 0.999589 | 0.000411 | 0.921475 | 0.078525 | 0.998450 | 0.001550 |
| Germany | 0.980223 | 0.019777 | 0.252942 | 0.747058 | 0.913297 | 0.086703 |
| Hungary | 1.000000 | 0.000000 | 0.579298 | 0.420702 | 0.999706 | 0.000294 |
| Iceland | 1.000000 | 0.000000 | 0.681346 | 0.318654 | 0.999928 | 0.000072 |
| India | 0.735031 | 0.264969 | 0.498494 | 0.501506 | 0.627660 | 0.372340 |
| Ireland | 0.909957 | 0.090043 | 0.299219 | 0.700781 | 0.854612 | 0.145388 |
| Isle of Man | 0.939151 | 0.060849 | 0.208853 | 0.791147 | 0.772205 | 0.227795 |
| Israel | 0.979081 | 0.020919 | 0.147715 | 0.852285 | 0.641781 | 0.358219 |
| Italy | 0.977181 | 0.022819 | 0.310784 | 0.689216 | 0.933415 | 0.066585 |
| Japan | 0.987240 | 0.012760 | 0.270076 | 0.729924 | 0.741184 | 0.258816 |
| Kazakhstan | 1.000000 | 0.000000 | 0.767972 | 0.232028 | 0.998609 | 0.001391 |
| Mexico | 1.000000 | 0.000000 | 0.982802 | 0.017198 | 0.999916 | 0.000084 |
| Netherlands | 0.999725 | 0.000275 | 0.818624 | 0.181376 | 0.998628 | 0.001372 |
| Nicaragua | 0.969301 | 0.030699 | 0.943645 | 0.056355 | 0.956541 | 0.043459 |
| Nigeria | 0.373004 | 0.626996 | 0.287528 | 0.712472 | 0.337431 | 0.662569 |
| Norway | 0.999975 | 0.000025 | 0.415353 | 0.584647 | 0.998319 | 0.001681 |
| Peru | 1.000000 | 0.000000 | 0.999974 | 0.000026 | 1.000000 | 0.000000 |
| Spain | 0.996394 | 0.003606 | 0.509978 | 0.490022 | 0.976501 | 0.023499 |
| Sri Lanka | 1.000000 | 0.000000 | 0.999327 | 0.000673 | 1.000000 | 0.000000 |
| Sweden | 1.000000 | 0.000000 | 0.913266 | 0.086734 | 1.000000 | 0.000000 |
| Switzerland | 0.999569 | 0.000431 | 0.486064 | 0.513936 | 0.998553 | 0.001447 |
| Uganda | 0.736495 | 0.263505 | 0.396778 | 0.603222 | 0.574877 | 0.425123 |
| Uruguay | 0.998907 | 0.001093 | 0.559124 | 0.440876 | 0.964207 | 0.035793 |
| USA 1 | 0.996419 | 0.003581 | 0.382127 | 0.617873 | 0.897229 | 0.102771 |
| USA 2 | 0.998940 | 0.001060 | 0.757792 | 0.242208 | 0.977713 | 0.022287 |
| USA 3 | 0.999197 | 0.000803 | 0.484911 | 0.515089 | 0.965185 | 0.034815 |

Table 15. Proportion estimates for the decomposition of alcohol Population Attributable Fraction (PAF) for pancreatitis consisting of drinkers that drink ≤ 120 g/day and > 120 g/day using a continuous model (Gamma, Log-Normal, and Weibull) for women

|  | Gamma Model | | Log Normal Model | | Weibull Model | |
| --- | --- | --- | --- | --- | --- | --- |
| Country | ≤ 120 g/day | > 120 g/day | ≤ 120 g/day | > 120 g/day | ≤ 120 g/day | > 120 g/day |
| Argentina | 0.999984 | 0.000016 | 0.638573 | 0.361427 | 0.993079 | 0.006921 |
| Australia | 0.999040 | 0.000960 | 0.374055 | 0.625945 | 0.955750 | 0.044250 |
| Australia 1 | 0.999972 | 0.000028 | 0.523274 | 0.476726 | 0.991750 | 0.008250 |
| Austria | 0.999950 | 0.000050 | 0.950867 | 0.049133 | 0.999836 | 0.000164 |
| Belize | 0.999367 | 0.000633 | 0.902223 | 0.097777 | 0.990039 | 0.009961 |
| Brazil | 0.784596 | 0.215404 | 0.452460 | 0.547540 | 0.637956 | 0.362044 |
| Canada | 0.999797 | 0.000203 | 0.628602 | 0.371398 | 0.995576 | 0.004424 |
| Costa Rica | 1.000000 | 0.000000 | 0.893974 | 0.106026 | 0.999222 | 0.000778 |
| Czech Republic | 0.980587 | 0.019413 | 0.381388 | 0.618612 | 0.866659 | 0.133341 |
| Denmark | 0.999656 | 0.000344 | 0.407876 | 0.592124 | 0.998531 | 0.001469 |
| ECAS: Finland | 1.000000 | 0.000000 | 0.552950 | 0.447050 | 0.999832 | 0.000168 |
| ECAS: France | 0.997056 | 0.002944 | 0.284998 | 0.715002 | 0.942277 | 0.057723 |
| ECAS: Germany | 0.999430 | 0.000570 | 0.398879 | 0.601121 | 0.986994 | 0.013006 |
| ECAS: Italy | 0.950439 | 0.049561 | 0.237958 | 0.762042 | 0.804872 | 0.195128 |
| ECAS: Sweden | 1.000000 | 0.000000 | 0.645829 | 0.354171 | 1.000000 | 0.000000 |
| ECAS: UK | 0.871182 | 0.128818 | 0.245213 | 0.754787 | 0.714379 | 0.285621 |
| Finland | 1.000000 | 0.000000 | 0.377836 | 0.622164 | 0.998706 | 0.001294 |
| France | 0.999921 | 0.000079 | 0.936047 | 0.063953 | 0.999531 | 0.000469 |
| Germany | 0.993423 | 0.006577 | 0.333727 | 0.666273 | 0.952790 | 0.047210 |
| Hungary | 1.000000 | 0.000000 | 0.623363 | 0.376637 | 0.999890 | 0.000110 |
| Iceland | 1.000000 | 0.000000 | 0.728281 | 0.271719 | 0.999976 | 0.000024 |
| India | 0.783059 | 0.216941 | 0.533430 | 0.466570 | 0.669488 | 0.330512 |
| Ireland | 0.957310 | 0.042690 | 0.383862 | 0.616138 | 0.918834 | 0.081166 |
| Isle of Man | 0.969683 | 0.030317 | 0.273814 | 0.726186 | 0.836580 | 0.163420 |
| Israel | 0.993292 | 0.006708 | 0.223822 | 0.776178 | 0.735278 | 0.264722 |
| Italy | 0.991766 | 0.008234 | 0.388879 | 0.611121 | 0.965480 | 0.034520 |
| Japan | 0.994994 | 0.005006 | 0.329104 | 0.670896 | 0.793752 | 0.206248 |
| Kazakhstan | 1.000000 | 0.000000 | 0.790716 | 0.209284 | 0.999229 | 0.000771 |
| Mexico | 1.000000 | 0.000000 | 0.985205 | 0.014795 | 0.999958 | 0.000042 |
| Netherlands | 0.999964 | 0.000036 | 0.850156 | 0.149844 | 0.999645 | 0.000355 |
| Nicaragua | 0.978690 | 0.021310 | 0.950119 | 0.049881 | 0.964675 | 0.035325 |
| Nigeria | 0.444819 | 0.555181 | 0.332187 | 0.667813 | 0.396174 | 0.603826 |
| Norway | 1.000000 | 0.000000 | 0.480070 | 0.519930 | 0.999456 | 0.000544 |
| Peru | 1.000000 | 0.000000 | 0.999983 | 0.000017 | 1.000000 | 0.000000 |
| Spain | 0.998685 | 0.001315 | 0.552536 | 0.447464 | 0.985444 | 0.014556 |
| Sri Lanka | 1.000000 | 0.000000 | 0.999428 | 0.000572 | 1.000000 | 0.000000 |
| Sweden | 1.000000 | 0.000000 | 0.928333 | 0.071667 | 1.000000 | 0.000000 |
| Switzerland | 0.999949 | 0.000051 | 0.557781 | 0.442219 | 0.999701 | 0.000299 |
| Uganda | 0.796986 | 0.203014 | 0.441705 | 0.558295 | 0.629193 | 0.370807 |
| Uruguay | 0.999693 | 0.000307 | 0.599301 | 0.400699 | 0.974936 | 0.025064 |
| USA 1 | 0.998865 | 0.001135 | 0.436459 | 0.563541 | 0.924527 | 0.075473 |
| USA 2 | 0.999711 | 0.000289 | 0.785001 | 0.214999 | 0.985165 | 0.014835 |
| USA 3 | 0.999839 | 0.000161 | 0.540672 | 0.459328 | 0.978393 | 0.021607 |

Table 16. Proportion estimates for the decomposition of alcohol Population Attributable Fraction (PAF) for pancreatitis consisting of drinkers that drink ≤ 150 g/day and > 150 g/day using a continuous model (Gamma, Log-Normal, and Weibull) for women

|  | Gamma Model | | Log Normal Model | | Weibull Model | |
| --- | --- | --- | --- | --- | --- | --- |
| Country | ≤ 150 g/day | > 150 g/day | ≤ 150 g/day | > 150 g/day | ≤ 150 g/day | > 150 g/day |
| Argentina | 1.000000 | 0.000000 | 0.722299 | 0.277701 | 0.997043 | 0.002957 |
| Australia | 0.999855 | 0.000145 | 0.516596 | 0.483404 | 0.980059 | 0.019941 |
| Australia 1 | 1.000000 | 0.000000 | 0.652327 | 0.347673 | 0.997312 | 0.002688 |
| Austria | 0.999993 | 0.000007 | 0.971095 | 0.028905 | 0.999986 | 0.000014 |
| Belize | 0.999878 | 0.000122 | 0.929255 | 0.070745 | 0.995349 | 0.004651 |
| Brazil | 0.878928 | 0.121072 | 0.563751 | 0.436249 | 0.745741 | 0.254259 |
| Canada | 0.999975 | 0.000025 | 0.732935 | 0.267065 | 0.998770 | 0.001230 |
| Costa Rica | 1.000000 | 0.000000 | 0.920760 | 0.079240 | 0.999697 | 0.000303 |
| Czech Republic | 0.994604 | 0.005396 | 0.527584 | 0.472416 | 0.931986 | 0.068014 |
| Denmark | 0.999960 | 0.000040 | 0.568856 | 0.431144 | 0.999762 | 0.000238 |
| ECAS: Finland | 1.000000 | 0.000000 | 0.704373 | 0.295627 | 1.000000 | 0.000000 |
| ECAS: France | 0.999599 | 0.000401 | 0.469870 | 0.530130 | 0.979953 | 0.020047 |
| ECAS: Germany | 0.999929 | 0.000071 | 0.577727 | 0.422273 | 0.996814 | 0.003186 |
| ECAS: Italy | 0.986689 | 0.013311 | 0.421648 | 0.578352 | 0.913354 | 0.086646 |
| ECAS: Sweden | 1.000000 | 0.000000 | 0.772962 | 0.227038 | 1.000000 | 0.000000 |
| ECAS: UK | 0.955855 | 0.044145 | 0.431874 | 0.568126 | 0.862943 | 0.137057 |
| Finland | 1.000000 | 0.000000 | 0.536113 | 0.463887 | 0.999726 | 0.000274 |
| France | 0.999994 | 0.000006 | 0.959492 | 0.040508 | 0.999922 | 0.000078 |
| Germany | 0.998837 | 0.001163 | 0.507900 | 0.492100 | 0.983911 | 0.016089 |
| Hungary | 1.000000 | 0.000000 | 0.719183 | 0.280817 | 0.999963 | 0.000037 |
| Iceland | 1.000000 | 0.000000 | 0.814230 | 0.185770 | 1.000000 | 0.000000 |
| India | 0.868280 | 0.131720 | 0.625301 | 0.374699 | 0.760981 | 0.239019 |
| Ireland | 0.988453 | 0.011547 | 0.557491 | 0.442509 | 0.972345 | 0.027655 |
| Isle of Man | 0.991170 | 0.008830 | 0.432863 | 0.567137 | 0.918057 | 0.081943 |
| Israel | 0.998863 | 0.001137 | 0.402603 | 0.597397 | 0.858638 | 0.141362 |
| Italy | 0.998394 | 0.001606 | 0.553643 | 0.446357 | 0.989083 | 0.010917 |
| Japan | 0.998921 | 0.001079 | 0.474103 | 0.525897 | 0.876061 | 0.123939 |
| Kazakhstan | 1.000000 | 0.000000 | 0.841808 | 0.158192 | 0.999718 | 0.000282 |
| Mexico | 1.000000 | 0.000000 | 0.989709 | 0.010291 | 0.999986 | 0.000014 |
| Netherlands | 1.000000 | 0.000000 | 0.902863 | 0.097137 | 0.999953 | 0.000047 |
| Nicaragua | 0.990171 | 0.009829 | 0.963639 | 0.036361 | 0.978207 | 0.021793 |
| Nigeria | 0.606549 | 0.393451 | 0.455039 | 0.544961 | 0.539583 | 0.460417 |
| Norway | 1.000000 | 0.000000 | 0.617577 | 0.382423 | 0.999901 | 0.000099 |
| Peru | 1.000000 | 0.000000 | 0.999991 | 0.000009 | 1.000000 | 0.000000 |
| Spain | 0.999741 | 0.000259 | 0.654114 | 0.345886 | 0.994078 | 0.005922 |
| Sri Lanka | 1.000000 | 0.000000 | 0.999596 | 0.000404 | 1.000000 | 0.000000 |
| Sweden | 1.000000 | 0.000000 | 0.953357 | 0.046643 | 1.000000 | 0.000000 |
| Switzerland | 1.000000 | 0.000000 | 0.693551 | 0.306449 | 0.999975 | 0.000025 |
| Uganda | 0.889243 | 0.110757 | 0.556425 | 0.443575 | 0.740509 | 0.259491 |
| Uruguay | 0.999959 | 0.000041 | 0.692863 | 0.307137 | 0.987668 | 0.012332 |
| USA 1 | 0.999811 | 0.000189 | 0.565064 | 0.434936 | 0.960170 | 0.039830 |
| USA 2 | 0.999952 | 0.000048 | 0.842324 | 0.157676 | 0.993208 | 0.006792 |
| USA 3 | 1.000000 | 0.000000 | 0.660300 | 0.339700 | 0.990992 | 0.009008 |

Table 17. Proportion estimates for the decomposition of alcohol Population Attributable Fraction (PAF) for pancreatitis consisting of drinkers that drink ≤ 200 g/day and > 200 g/day using a continuous model (Gamma, Log-Normal, and Weibull) for women

|  | Gamma Model | | Log Normal Model | | Weibull Model | |
| --- | --- | --- | --- | --- | --- | --- |
| Country | ≤ 200 g/day | > 200 g/day | ≤ 200 g/day | > 200 g/day | ≤ 200 g/day | > 200 g/day |
| Argentina | 1.000000 | 0.000000 | 0.870444 | 0.129556 | 0.999613 | 0.000387 |
| Australia | 1.000000 | 0.000000 | 0.772223 | 0.227777 | 0.997166 | 0.002834 |
| Australia 1 | 1.000000 | 0.000000 | 0.854379 | 0.145621 | 0.999814 | 0.000186 |
| Austria | 1.000000 | 0.000000 | 0.992170 | 0.007830 | 1.000000 | 0.000000 |
| Belize | 1.000000 | 0.000000 | 0.970855 | 0.029145 | 0.999285 | 0.000715 |
| Brazil | 0.974681 | 0.025319 | 0.780529 | 0.219471 | 0.905077 | 0.094923 |
| Canada | 1.000000 | 0.000000 | 0.891522 | 0.108478 | 0.999938 | 0.000062 |
| Costa Rica | 1.000000 | 0.000000 | 0.965053 | 0.034947 | 0.999966 | 0.000034 |
| Czech Republic | 0.999770 | 0.000230 | 0.782793 | 0.217207 | 0.987552 | 0.012448 |
| Denmark | 1.000000 | 0.000000 | 0.820590 | 0.179410 | 1.000000 | 0.000000 |
| ECAS: Finland | 1.000000 | 0.000000 | 0.898402 | 0.101598 | 1.000000 | 0.000000 |
| ECAS: France | 1.000000 | 0.000000 | 0.770679 | 0.229321 | 0.998388 | 0.001612 |
| ECAS: Germany | 1.000000 | 0.000000 | 0.836374 | 0.163626 | 0.999870 | 0.000130 |
| ECAS: Italy | 0.999478 | 0.000522 | 0.737772 | 0.262228 | 0.988574 | 0.011426 |
| ECAS: Sweden | 1.000000 | 0.000000 | 0.926476 | 0.073524 | 1.000000 | 0.000000 |
| ECAS: UK | 0.997078 | 0.002922 | 0.747137 | 0.252863 | 0.978907 | 0.021093 |
| Finland | 1.000000 | 0.000000 | 0.796739 | 0.203261 | 1.000000 | 0.000000 |
| France | 1.000000 | 0.000000 | 0.987270 | 0.012730 | 1.000000 | 0.000000 |
| Germany | 0.999978 | 0.000022 | 0.789084 | 0.210916 | 0.998821 | 0.001179 |
| Hungary | 1.000000 | 0.000000 | 0.876656 | 0.123344 | 1.000000 | 0.000000 |
| Iceland | 1.000000 | 0.000000 | 0.931735 | 0.068265 | 1.000000 | 0.000000 |
| India | 0.966938 | 0.033062 | 0.808280 | 0.191720 | 0.904904 | 0.095096 |
| Ireland | 0.999544 | 0.000456 | 0.821662 | 0.178338 | 0.998117 | 0.001883 |
| Isle of Man | 0.999582 | 0.000418 | 0.726693 | 0.273307 | 0.985682 | 0.014318 |
| Israel | 1.000000 | 0.000000 | 0.720208 | 0.279792 | 0.970346 | 0.029654 |
| Italy | 0.999963 | 0.000037 | 0.813209 | 0.186791 | 0.999331 | 0.000669 |
| Japan | 0.999980 | 0.000020 | 0.744045 | 0.255955 | 0.966547 | 0.033453 |
| Kazakhstan | 1.000000 | 0.000000 | 0.928579 | 0.071421 | 0.999972 | 0.000028 |
| Mexico | 1.000000 | 0.000000 | 0.996063 | 0.003937 | 1.000000 | 0.000000 |
| Netherlands | 1.000000 | 0.000000 | 0.967992 | 0.032008 | 1.000000 | 0.000000 |
| Nicaragua | 0.998706 | 0.001294 | 0.984809 | 0.015191 | 0.993874 | 0.006126 |
| Nigeria | 0.856708 | 0.143292 | 0.711386 | 0.288614 | 0.794171 | 0.205829 |
| Norway | 1.000000 | 0.000000 | 0.837089 | 0.162911 | 1.000000 | 0.000000 |
| Peru | 1.000000 | 0.000000 | 1.000000 | 0.000000 | 1.000000 | 0.000000 |
| Spain | 0.999993 | 0.000007 | 0.836873 | 0.163127 | 0.999345 | 0.000655 |
| Sri Lanka | 1.000000 | 0.000000 | 0.999865 | 0.000135 | 1.000000 | 0.000000 |
| Sweden | 1.000000 | 0.000000 | 0.984412 | 0.015588 | 1.000000 | 0.000000 |
| Switzerland | 1.000000 | 0.000000 | 0.884669 | 0.115331 | 1.000000 | 0.000000 |
| Uganda | 0.978401 | 0.021599 | 0.778147 | 0.221853 | 0.903748 | 0.096252 |
| Uruguay | 1.000000 | 0.000000 | 0.857434 | 0.142566 | 0.997850 | 0.002150 |
| USA 1 | 1.000000 | 0.000000 | 0.795201 | 0.204799 | 0.991922 | 0.008078 |
| USA 2 | 1.000000 | 0.000000 | 0.933180 | 0.066820 | 0.998986 | 0.001014 |
| USA 3 | 1.000000 | 0.000000 | 0.853517 | 0.146483 | 0.998904 | 0.001096 |

Table 18. Proportion estimates for the decomposition of alcohol Population Attributable Fraction (PAF) for pancreatitis consisting of drinkers that drink ≤ 96 g/day and > 96 g/day using a continuous model (Gamma, Log-Normal, and Weibull) for men

|  | Gamma Model | | Log Normal Model | | Weibull Model | |
| --- | --- | --- | --- | --- | --- | --- |
| Country | ≤ 96 g/day | > 96 g/day | ≤ 96 g/day | > 96 g/day | ≤ 96 g/day | > 96 g/day |
| Argentina | 0.590879 | 0.409121 | 0.134609 | 0.865391 | 0.438151 | 0.561849 |
| Australia | 0.823029 | 0.176971 | 0.146240 | 0.853760 | 0.671679 | 0.328321 |
| Austria | 0.650217 | 0.349783 | 0.297569 | 0.702431 | 0.639035 | 0.360965 |
| Belize | 0.420631 | 0.579369 | 0.227197 | 0.772803 | 0.345403 | 0.654597 |
| Brazil | 0.331062 | 0.668938 | 0.172587 | 0.827413 | 0.258551 | 0.741449 |
| Canada | 0.721921 | 0.278079 | 0.204317 | 0.795683 | 0.591947 | 0.408053 |
| Costa Rica | 0.797841 | 0.202159 | 0.225525 | 0.774475 | 0.545344 | 0.454656 |
| Czech Republic | 0.263754 | 0.736246 | 0.108922 | 0.891078 | 0.235426 | 0.764574 |
| Denmark | 0.766800 | 0.233200 | 0.198406 | 0.801594 | 0.717426 | 0.282574 |
| ECAS: Finland | 0.814137 | 0.185863 | 0.225841 | 0.774159 | 0.750194 | 0.249806 |
| ECAS: France | 0.495663 | 0.504337 | 0.129568 | 0.870432 | 0.400876 | 0.599124 |
| ECAS: Germany | 0.847596 | 0.152404 | 0.191253 | 0.808747 | 0.756038 | 0.243962 |
| ECAS: Italy | 0.665680 | 0.334320 | 0.142681 | 0.857319 | 0.628510 | 0.371490 |
| ECAS: Sweden | 0.938876 | 0.061124 | 0.244911 | 0.755089 | 0.869387 | 0.130613 |
| ECAS: UK | 0.234510 | 0.765490 | 0.118758 | 0.881242 | 0.211996 | 0.788004 |
| Finland | 0.857554 | 0.142446 | 0.169034 | 0.830966 | 0.746769 | 0.253231 |
| France | 0.563971 | 0.436029 | 0.222519 | 0.777481 | 0.502307 | 0.497693 |
| Germany | 0.650399 | 0.349601 | 0.154000 | 0.846000 | 0.601184 | 0.398816 |
| Hungary | 0.886791 | 0.113209 | 0.144701 | 0.855299 | 0.626514 | 0.373486 |
| Iceland | 0.995283 | 0.004717 | 0.354648 | 0.645352 | 0.990496 | 0.009504 |
| India | 0.157518 | 0.842482 | 0.115256 | 0.884744 | 0.144008 | 0.855992 |
| Ireland | 0.225293 | 0.774707 | 0.136703 | 0.863297 | 0.207931 | 0.792069 |
| Isle of Man | 0.281400 | 0.718600 | 0.099344 | 0.900656 | 0.225825 | 0.774175 |
| Israel | 0.702207 | 0.297793 | 0.122373 | 0.877627 | 0.390596 | 0.609404 |
| Italy | 0.613424 | 0.386576 | 0.158772 | 0.841228 | 0.627089 | 0.372911 |
| Japan | 0.333772 | 0.666228 | 0.086897 | 0.913103 | 0.210845 | 0.789155 |
| Kazakhstan | 0.394740 | 0.605260 | 0.155607 | 0.844393 | 0.306158 | 0.693842 |
| Mexico | 0.470241 | 0.529759 | 0.151479 | 0.848521 | 0.303521 | 0.696479 |
| Netherlands | 0.814195 | 0.185805 | 0.261467 | 0.738533 | 0.781442 | 0.218558 |
| Nicaragua | 0.489623 | 0.510377 | 0.317135 | 0.682865 | 0.420987 | 0.579013 |
| Nigeria | 0.206814 | 0.793186 | 0.130135 | 0.869865 | 0.186008 | 0.813992 |
| Norway | 0.901518 | 0.098482 | 0.209879 | 0.790121 | 0.780955 | 0.219045 |
| Peru | 0.999220 | 0.000780 | 0.726406 | 0.273594 | 0.990678 | 0.009322 |
| Spain | 0.609787 | 0.390213 | 0.179199 | 0.820801 | 0.546576 | 0.453424 |
| Sri Lanka | 0.269248 | 0.730752 | 0.177106 | 0.822894 | 0.224118 | 0.775882 |
| Sweden | 0.999403 | 0.000597 | 0.413939 | 0.586061 | 0.994767 | 0.005233 |
| Switzerland | 0.775149 | 0.224851 | 0.249798 | 0.750202 | 0.704176 | 0.295824 |
| Uganda | 0.137701 | 0.862299 | 0.105137 | 0.894863 | 0.132215 | 0.867785 |
| Uruguay | 0.412697 | 0.587303 | 0.137022 | 0.862978 | 0.291961 | 0.708039 |
| USA 2 | 0.678260 | 0.321740 | 0.202518 | 0.797482 | 0.462832 | 0.537168 |
| USA 3 | 0.549735 | 0.450265 | 0.151656 | 0.848344 | 0.353351 | 0.646649 |

Table 19. Proportion estimates for the decomposition of alcohol Population Attributable Fraction (PAF) for pancreatitis consisting of drinkers that drink ≤ 120 g/day and > 120 g/day using a continuous model (Gamma, Log-Normal, and Weibull) for men

|  | Gamma Model | | Log Normal Model | | Weibull Model | |
| --- | --- | --- | --- | --- | --- | --- |
| Country | ≤ 120 g/day | > 120 g/day | ≤ 120 g/day | > 120 g/day | ≤ 120 g/day | > 120 g/day |
| Argentina | 0.718332 | 0.281668 | 0.205686 | 0.794314 | 0.560865 | 0.439135 |
| Australia | 0.901195 | 0.098805 | 0.218484 | 0.781516 | 0.773363 | 0.226637 |
| Austria | 0.779303 | 0.220697 | 0.396630 | 0.603370 | 0.775002 | 0.224998 |
| Belize | 0.533805 | 0.466195 | 0.293851 | 0.706149 | 0.440847 | 0.559153 |
| Brazil | 0.438042 | 0.561958 | 0.232071 | 0.767929 | 0.341462 | 0.658538 |
| Canada | 0.824639 | 0.175361 | 0.282523 | 0.717477 | 0.703914 | 0.296086 |
| Costa Rica | 0.869125 | 0.130875 | 0.288664 | 0.711336 | 0.630011 | 0.369989 |
| Czech Republic | 0.395533 | 0.604467 | 0.178374 | 0.821626 | 0.355722 | 0.644278 |
| Denmark | 0.869293 | 0.130707 | 0.287830 | 0.712170 | 0.830007 | 0.169993 |
| ECAS: Finland | 0.903552 | 0.096448 | 0.320760 | 0.679240 | 0.855818 | 0.144182 |
| ECAS: France | 0.642576 | 0.357424 | 0.207170 | 0.792830 | 0.539273 | 0.460727 |
| ECAS: Germany | 0.923813 | 0.076187 | 0.279637 | 0.720363 | 0.854550 | 0.145450 |
| ECAS: Italy | 0.792796 | 0.207204 | 0.223623 | 0.776377 | 0.758195 | 0.241805 |
| ECAS: Sweden | 0.976221 | 0.023779 | 0.338559 | 0.661441 | 0.933107 | 0.066893 |
| ECAS: UK | 0.368478 | 0.631522 | 0.196581 | 0.803419 | 0.336530 | 0.663470 |
| Finland | 0.926689 | 0.073311 | 0.248083 | 0.751917 | 0.839840 | 0.160160 |
| France | 0.692620 | 0.307380 | 0.304462 | 0.695538 | 0.629234 | 0.370766 |
| Germany | 0.778396 | 0.221604 | 0.235458 | 0.764542 | 0.732295 | 0.267705 |
| Hungary | 0.943249 | 0.056751 | 0.217292 | 0.782708 | 0.727594 | 0.272406 |
| Iceland | 0.998970 | 0.001030 | 0.439449 | 0.560551 | 0.997300 | 0.002700 |
| India | 0.247315 | 0.752685 | 0.174803 | 0.825197 | 0.221427 | 0.778573 |
| Ireland | 0.354763 | 0.645237 | 0.217690 | 0.782310 | 0.330615 | 0.669385 |
| Isle of Man | 0.413277 | 0.586723 | 0.164778 | 0.835222 | 0.338179 | 0.661821 |
| Israel | 0.815137 | 0.184863 | 0.195215 | 0.804785 | 0.510074 | 0.489926 |
| Italy | 0.748614 | 0.251386 | 0.241731 | 0.758269 | 0.760142 | 0.239858 |
| Japan | 0.468993 | 0.531007 | 0.147695 | 0.852305 | 0.314308 | 0.685692 |
| Kazakhstan | 0.517920 | 0.482080 | 0.221411 | 0.778589 | 0.407927 | 0.592073 |
| Mexico | 0.595011 | 0.404989 | 0.217473 | 0.782527 | 0.402727 | 0.597273 |
| Netherlands | 0.898872 | 0.101128 | 0.351446 | 0.648554 | 0.874264 | 0.125736 |
| Nicaragua | 0.587478 | 0.412522 | 0.377805 | 0.622195 | 0.503798 | 0.496202 |
| Nigeria | 0.306592 | 0.693408 | 0.190844 | 0.809156 | 0.273254 | 0.726746 |
| Norway | 0.953170 | 0.046830 | 0.292149 | 0.707851 | 0.862918 | 0.137082 |
| Peru | 0.999858 | 0.000142 | 0.773130 | 0.226870 | 0.996308 | 0.003692 |
| Spain | 0.726885 | 0.273115 | 0.250317 | 0.749683 | 0.662604 | 0.337396 |
| Sri Lanka | 0.366065 | 0.633935 | 0.233995 | 0.766005 | 0.297963 | 0.702037 |
| Sweden | 0.999925 | 0.000075 | 0.493311 | 0.506689 | 0.998423 | 0.001577 |
| Switzerland | 0.875446 | 0.124554 | 0.345109 | 0.654891 | 0.819958 | 0.180042 |
| Uganda | 0.224113 | 0.775887 | 0.164615 | 0.835385 | 0.211721 | 0.788279 |
| Uruguay | 0.541527 | 0.458473 | 0.203266 | 0.796734 | 0.396554 | 0.603446 |
| USA 2 | 0.779628 | 0.220372 | 0.271303 | 0.728697 | 0.562686 | 0.437314 |
| USA 3 | 0.675640 | 0.324360 | 0.221716 | 0.778284 | 0.461331 | 0.538669 |

Table 20. Proportion estimates for the decomposition of alcohol Population Attributable Fraction (PAF) for pancreatitis consisting of drinkers that drink ≤ 150 g/day and > 150 g/day using a continuous model (Gamma, Log-Normal, and Weibull) for men

|  | Gamma Model | | Log Normal Model | | Weibull Model | |
| --- | --- | --- | --- | --- | --- | --- |
| Country | ≤ 150 g/day | > 150 g/day | ≤ 150 g/day | > 150 g/day | ≤ 150 g/day | > 150 g/day |
| Argentina | 0.872366 | 0.127634 | 0.380184 | 0.619816 | 0.750406 | 0.249594 |
| Australia | 0.966729 | 0.033271 | 0.393346 | 0.606654 | 0.893975 | 0.106025 |
| Austria | 0.913584 | 0.086416 | 0.586301 | 0.413699 | 0.914932 | 0.085068 |
| Belize | 0.726727 | 0.273273 | 0.454199 | 0.545801 | 0.627338 | 0.372662 |
| Brazil | 0.642040 | 0.357960 | 0.386552 | 0.613448 | 0.523773 | 0.476227 |
| Canada | 0.930327 | 0.069673 | 0.459702 | 0.540298 | 0.851470 | 0.148530 |
| Costa Rica | 0.945644 | 0.054356 | 0.443722 | 0.556278 | 0.772193 | 0.227807 |
| Czech Republic | 0.633540 | 0.366460 | 0.353612 | 0.646388 | 0.585767 | 0.414233 |
| Denmark | 0.956147 | 0.043853 | 0.479110 | 0.520890 | 0.936670 | 0.063330 |
| ECAS: Finland | 0.970773 | 0.029227 | 0.514392 | 0.485608 | 0.949147 | 0.050853 |
| ECAS: France | 0.831096 | 0.168904 | 0.391278 | 0.608722 | 0.747185 | 0.252815 |
| ECAS: Germany | 0.977904 | 0.022096 | 0.469980 | 0.530020 | 0.945483 | 0.054517 |
| ECAS: Italy | 0.919955 | 0.080045 | 0.410802 | 0.589198 | 0.898680 | 0.101320 |
| ECAS: Sweden | 0.994946 | 0.005054 | 0.527744 | 0.472256 | 0.979430 | 0.020570 |
| ECAS: UK | 0.614639 | 0.385361 | 0.383171 | 0.616829 | 0.576291 | 0.423709 |
| Finland | 0.977875 | 0.022125 | 0.429667 | 0.570333 | 0.934334 | 0.065666 |
| France | 0.856385 | 0.143615 | 0.484868 | 0.515132 | 0.808715 | 0.191285 |
| Germany | 0.911619 | 0.088381 | 0.422214 | 0.577786 | 0.882616 | 0.117384 |
| Hungary | 0.983340 | 0.016660 | 0.392373 | 0.607627 | 0.859626 | 0.140374 |
| Iceland | 0.999886 | 0.000114 | 0.605499 | 0.394501 | 0.999598 | 0.000402 |
| India | 0.456691 | 0.543309 | 0.334175 | 0.665825 | 0.409949 | 0.590051 |
| Ireland | 0.599860 | 0.400140 | 0.407279 | 0.592721 | 0.571415 | 0.428585 |
| Isle of Man | 0.647052 | 0.352948 | 0.334435 | 0.665565 | 0.559589 | 0.440411 |
| Israel | 0.927668 | 0.072332 | 0.372621 | 0.627379 | 0.704183 | 0.295817 |
| Italy | 0.896192 | 0.103808 | 0.430146 | 0.569854 | 0.902478 | 0.097522 |
| Japan | 0.693366 | 0.306634 | 0.310763 | 0.689237 | 0.526067 | 0.473933 |
| Kazakhstan | 0.722290 | 0.277710 | 0.386729 | 0.613271 | 0.605439 | 0.394561 |
| Mexico | 0.782231 | 0.217769 | 0.383045 | 0.616955 | 0.596309 | 0.403691 |
| Netherlands | 0.967394 | 0.032606 | 0.535711 | 0.464289 | 0.956087 | 0.043913 |
| Nicaragua | 0.756158 | 0.243842 | 0.521817 | 0.478183 | 0.667006 | 0.332994 |
| Nigeria | 0.522985 | 0.477015 | 0.351120 | 0.648880 | 0.472319 | 0.527681 |
| Norway | 0.987213 | 0.012787 | 0.473160 | 0.526840 | 0.944347 | 0.055653 |
| Peru | 1.000000 | 0.000000 | 0.851885 | 0.148115 | 0.999146 | 0.000854 |
| Spain | 0.873587 | 0.126413 | 0.421327 | 0.578673 | 0.825529 | 0.174471 |
| Sri Lanka | 0.569128 | 0.430872 | 0.384063 | 0.615937 | 0.471647 | 0.528353 |
| Sweden | 1.000000 | 0.000000 | 0.645732 | 0.354268 | 0.999749 | 0.000251 |
| Switzerland | 0.958946 | 0.041054 | 0.536388 | 0.463612 | 0.932021 | 0.067979 |
| Uganda | 0.432057 | 0.567943 | 0.325061 | 0.674939 | 0.406632 | 0.593368 |
| Uruguay | 0.744860 | 0.255140 | 0.370641 | 0.629359 | 0.598152 | 0.401848 |
| USA 2 | 0.900633 | 0.099367 | 0.436209 | 0.563791 | 0.731299 | 0.268701 |
| USA 3 | 0.841751 | 0.158249 | 0.392907 | 0.607093 | 0.654876 | 0.345124 |

Table 21. Proportion estimates for the decomposition of alcohol Population Attributable Fraction (PAF) for pancreatitis consisting of drinkers that drink ≤ 200 g/day and > 200 g/day using a continuous model (Gamma, Log-Normal, and Weibull) for men

|  | Gamma Model | | Log Normal Model | | Weibull Model | |
| --- | --- | --- | --- | --- | --- | --- |
| Country | ≤ 200 g/day | > 200 g/day | ≤ 200 g/day | > 200 g/day | ≤ 200 g/day | > 200 g/day |
| Argentina | 0.984062 | 0.015938 | 0.702360 | 0.297640 | 0.943782 | 0.056218 |
| Australia | 0.997877 | 0.002123 | 0.711742 | 0.288258 | 0.984590 | 0.015410 |
| Austria | 0.992586 | 0.007414 | 0.850008 | 0.149992 | 0.993628 | 0.006372 |
| Belize | 0.937446 | 0.062554 | 0.743408 | 0.256592 | 0.878892 | 0.121108 |
| Brazil | 0.899319 | 0.100681 | 0.689876 | 0.310124 | 0.811089 | 0.188911 |
| Canada | 0.993593 | 0.006407 | 0.759574 | 0.240426 | 0.975131 | 0.024869 |
| Costa Rica | 0.994385 | 0.005615 | 0.731146 | 0.268854 | 0.936790 | 0.063210 |
| Czech Republic | 0.909789 | 0.090211 | 0.684928 | 0.315072 | 0.879338 | 0.120662 |
| Denmark | 0.997275 | 0.002725 | 0.782406 | 0.217594 | 0.994916 | 0.005084 |
| ECAS: Finland | 0.998551 | 0.001449 | 0.806448 | 0.193552 | 0.996368 | 0.003632 |
| ECAS: France | 0.976907 | 0.023093 | 0.718039 | 0.281961 | 0.948548 | 0.051452 |
| ECAS: Germany | 0.998992 | 0.001008 | 0.775293 | 0.224707 | 0.995340 | 0.004660 |
| ECAS: Italy | 0.993145 | 0.006855 | 0.733834 | 0.266166 | 0.989235 | 0.010765 |
| ECAS: Sweden | 0.999876 | 0.000124 | 0.811673 | 0.188327 | 0.998857 | 0.001143 |
| ECAS: UK | 0.904220 | 0.095780 | 0.715460 | 0.284540 | 0.880238 | 0.119762 |
| Finland | 0.998895 | 0.001105 | 0.742088 | 0.257912 | 0.993004 | 0.006996 |
| France | 0.981016 | 0.018984 | 0.779332 | 0.220668 | 0.967095 | 0.032905 |
| Germany | 0.991874 | 0.008126 | 0.741381 | 0.258619 | 0.986156 | 0.013844 |
| Hungary | 0.999202 | 0.000798 | 0.710836 | 0.289164 | 0.973790 | 0.026210 |
| Iceland | 1.000000 | 0.000000 | 0.847071 | 0.152929 | 1.000000 | 0.000000 |
| India | 0.793521 | 0.206479 | 0.656404 | 0.343596 | 0.741121 | 0.258879 |
| Ireland | 0.897083 | 0.102917 | 0.734852 | 0.265148 | 0.879664 | 0.120336 |
| Isle of Man | 0.914193 | 0.085807 | 0.665856 | 0.334144 | 0.858479 | 0.141521 |
| Israel | 0.993461 | 0.006539 | 0.698736 | 0.301264 | 0.921181 | 0.078819 |
| Italy | 0.989719 | 0.010281 | 0.748218 | 0.251782 | 0.990665 | 0.009335 |
| Japan | 0.931936 | 0.068064 | 0.642126 | 0.357874 | 0.831117 | 0.168883 |
| Kazakhstan | 0.938882 | 0.061118 | 0.699515 | 0.300485 | 0.871219 | 0.128781 |
| Mexico | 0.959089 | 0.040911 | 0.696707 | 0.303293 | 0.862338 | 0.137662 |
| Netherlands | 0.998144 | 0.001856 | 0.814536 | 0.185464 | 0.996975 | 0.003025 |
| Nicaragua | 0.943021 | 0.056979 | 0.777886 | 0.222114 | 0.889868 | 0.110132 |
| Nigeria | 0.839476 | 0.160524 | 0.669903 | 0.330097 | 0.792219 | 0.207781 |
| Norway | 0.999481 | 0.000519 | 0.770913 | 0.229087 | 0.994114 | 0.005886 |
| Peru | 1.000000 | 0.000000 | 0.950392 | 0.049608 | 0.999977 | 0.000023 |
| Spain | 0.983585 | 0.016415 | 0.729002 | 0.270998 | 0.969478 | 0.030522 |
| Sri Lanka | 0.857901 | 0.142099 | 0.683988 | 0.316012 | 0.769539 | 0.230461 |
| Sweden | 1.000000 | 0.000000 | 0.864059 | 0.135941 | 1.000000 | 0.000000 |
| Switzerland | 0.997561 | 0.002439 | 0.819202 | 0.180798 | 0.994424 | 0.005576 |
| Uganda | 0.777557 | 0.222443 | 0.651170 | 0.348830 | 0.746364 | 0.253636 |
| Uruguay | 0.948187 | 0.051813 | 0.689569 | 0.310431 | 0.868537 | 0.131463 |
| USA 2 | 0.987645 | 0.012355 | 0.733815 | 0.266185 | 0.926290 | 0.073710 |
| USA 3 | 0.976347 | 0.023653 | 0.708288 | 0.291712 | 0.895672 | 0.104328 |
